# Targeted Sirtuin 3 Activation by Biomimetic Black Phosphorus Nanosheets Mitigates Sepsis-Induced Acute Kidney Injury through Yeast Mitochondrial Escape 1-Like 1 Deacetylation

**DOI:** 10.34133/bmr.0379

**Published:** 2026-06-29

**Authors:** Yiqiong Yang, Rui Zuo, Yi Wang, Rumeng Liu, Yi Zhou, Jun Wang

**Affiliations:** ^1^ Department of Emergency, Nanjing Drum Tower Hospital Clinical College of Nanjing Medical University, Nanjing 210000, China.; ^2^ Department of Emergency, Nanjing Drum Tower Hospital, The Affiliated Hospital of Nanjing University Medical School, Nanjing 210000, China.

## Abstract

Sepsis-induced acute kidney injury (AKI) is characterized by mitochondrial dysfunction and dysregulated inflammation, with a lack of effective therapies. Studies have found that down-regulation of Sirtuin 3 (Sirt3) expression in renal tubular epithelial cells is associated with mitochondrial imbalance, suggesting its potential as a therapeutic target. Based on this, the research team developed a targeted nanodelivery system: black phosphorus nanosheets loaded with a cortistatin agonist were encapsulated with macrophage membranes modified with (KKEEE)₃K peptides to specifically deliver Sirt3-activating components to the kidneys. This nanosystem demonstrated favorable stability and biocompatibility. Ex vivo experiments confirmed its ability to alleviate lipopolysaccharide-induced oxidative stress, apoptosis, and inflammation in HK-2 cells, while restoring mitochondrial function. Mechanistically, the nanomaterial regulates mitochondrial homeostasis by activating the Sirt3–YME1L1 deacetylation axis. This study provides a novel nano-therapeutic strategy for sepsis-induced AKI, combining targeting capability with metabolism regulation, and holds broad implications for the treatment of inflammatory organ damage.

## Introduction

Sepsis-induced acute kidney injury (SA-AKI) is a common and life-threatening complication in the intensive care unit [1–3], accounting for more than 50% of all AKI cases, with a mortality rate as high as 30%–50% [4–6]. Sepsis leads to renal tubular epithelial cell injury through multiple mechanisms, including systemic inflammatory response, oxidative stress, and mitochondrial dysfunction, ultimately resulting in renal failure [7–10]. Although advances have been made in supportive care, such as fluid resuscitation and renal replacement therapy, specific therapeutic strategies for SA-AKI remain limited [11–13]. There is an urgent need to develop novel treatments that simultaneously target inflammation and mitochondrial dysfunction. Mitochondria, as the cellular powerhouses, play a crucial role in maintaining tubular epithelial cell survival, with their dynamic balance—involving fission and fusion—being particularly important [14,15]. Studies have shown that the imbalance between the mitochondrial fission protein Drp1 and the fusion protein OPA1 is a key factor in apoptosis and inflammatory amplification in SA-AKI [16–18]. However, the molecular mechanisms governing mitochondrial dynamics in SA-AKI are not fully understood, and the role of deacetylation modifications in this process remains largely unexplored.

In recent years, Sirtuin 3 (Sirt3), an NAD^+^-dependent deacetylase, has gained increasing attention due to its central role in regulating mitochondrial function [19–22]. Sirt3 maintains oxidative phosphorylation efficiency and antioxidant defense capacity by deacetylating multiple mitochondrial proteins, such as Complex I, Complex II, and YME1L1 [23]. YME1L1 is an inner mitochondrial membrane protease involved in regulating mitochondrial cristae morphology and protein homeostasis, and its activity is modulated by acetylation [24,25]. In ischemia–reperfusion injury models, Sirt3 protects mitochondrial function by deacetylating YME1L1. However, whether this mechanism operates in SA-AKI remains unclear.

On the other hand, nanomaterial-based drug delivery systems offer new opportunities for SA-AKI treatment [26–31]. The emergence of 2-dimensional nanomaterials has brought opportunities for addressing and treating inflammatory-related diseases. Studies have demonstrated that molybdenum disulfide, MXene, and graphene derivatives exhibit broad applications in anti-inflammatory effects and organ injury treatment, achieving favorable therapeutic outcomes. This highlights the universal potential of 2-dimensional material families in biomedical applications [32,33]. Black phosphorus nanosheets (BPNSs) have emerged as promising drug carriers due to their excellent biocompatibility, high drug-loading capacity, and biodegradability [34–37]. Macrophage membrane coating can confer immune evasion and inflammation-targeting properties to nanoparticles, while targeting peptides such as (KKEEE)₃K can further enhance renal accumulation [38–42]. Nevertheless, no study has yet integrated BPNSs with Sirt3 agonists (e.g., corticosterone [CORT]) and targeting peptides to synergistically modulate mitochondrial function and treat SA-AKI. Although previous studies have explored the protective role of Sirt3 in kidney injury, several key scientific questions remain unresolved: Does Sirt3 regulate mitochondrial dynamics (Drp1 and OPA1 balance) in SA-AKI via deacetylation of YME1L1? Existing nanodelivery systems lack mitochondrial specificity and fail to integrate Sirt3 activation with inflammation regulation. Moreover, most studies focus solely on anti-inflammatory or antioxidant effects, overlooking the synergistic benefits of mitochondrial repair.

To address these issues, this study proposes the design and synthesis of a macrophage membrane-coated BPNSs loaded with CORT and modified with the targeting peptide (KKEEE)₃K, aiming to achieve kidney-specific delivery and Sirt3 activation. The stability, drug-loading efficiency, and biosafety of BPNSs@CORT@Raw264.7@(KKEEE)₃K will be verified using techniques such as transmission electron microscopy (TEM) and x-ray photoelectron spectroscopy (XPS). In a lipopolysaccharide (LPS)-induced HK-2 cell model, the effects of the nanocomposite on mitochondrial membrane potential (MMP; JC-1), reactive oxygen species (ROS) levels, apoptosis (flow cytometry), and inflammatory cytokines (enzyme-linked immunosorbent assay [ELISA]) will be evaluated. The Sirt3–YME1L1 interaction will be confirmed by co-immunoprecipitation (Co-IP). In a murine model of SA-AKI, the therapeutic efficacy of the nanomaterial will be assessed by improvements in renal function (serum creatinine and blood urea nitrogen) and renal pathology (hematoxylin and eosin [H&E] staining). By elucidating the regulatory role of the Sirt3–YME1L1 axis in mitochondrial fission/fusion in SA-AKI, this study aims to further suppress inflammation (via the nuclear factor-kappa B [NF-κB] pathway) and restore mitochondrial integrity (Drp1 and OPA1 balance).

Centered on the core hypothesis that “macrophage membrane-coated BPNSs loaded with CORT and modified with a targeting peptide can alleviate SA-AKI by activating the Sirt3–YME1L1 pathway”, this research seeks to clarify the novel mechanism by which the Sirt3–YME1L1 axis confers mitochondrial protection in SA-AKI. It may provide a new therapeutic target for interrupting the “inflammation–mitochondrial dysfunction” vicious cycle, while also developing a nanodrug with targeting capability, biocompatibility, and synergistic therapeutic functions, thereby offering a paradigm for nanomaterial-based treatment of sepsis-induced multiorgan injury.

## Materials and Methods

### Experimental materials

The following materials were used in this study: Cell culture flasks T25 (TCF012050), T75 (TCF012250), 6-well plates (TCP011006), 15-ml centrifuge tubes (CFT920150), 50-ml centrifuge tubes (CFT920500) (Jet BIOFIL, China), 1.5-ml enzyme-free centrifuge tubes (MCT-150-C) (KeyGEN, China), Transwell chambers (8-μm model: 3422) (Corning, China), Transwell chambers (3-μm model: 3415) (Corning, China), Transwell chambers (5-μm model: 3421) (Corning, China), Small animal surgical instrument set (FS500) (Beyotime, China), and 10-μl micropipette (Hamilton, Switzerland).

### Experimental reagents

The following reagents were used in this study: Fetal bovine serum (FSD500) (Excell Bio, China), penicillin–streptomycin solution (100×) (C0222) (Beyotime, China), Dulbecco’s Modified Eagle Medium (DMEM)/F12 Medium (11320033) (Gibco, USA), Protease (R001100) (Gibco, China), Cell Freeze-Liquid (C0210B-50 ml) and phosphate-buffered saline (PBS) Buffer (C0221A) (Beyotime, China), RNA-specific Transfection Reagent-RNAFit (HB-RF-1000, Hanheng Bio), CCK-8 Cell Proliferation Assay Kit (BA00208) (Bioss, China), 4% paraformaldehyde (P0099), Triton X-100 (P0096), 10% goat serum blocking solution (C01-03001) (Bioss, China), 1% Trypan Red (V5265-250 ml) (Sigma-Aldrich, USA), 1×EDTA antigen retrieval buffer (Beijing Suolebao Technology Co., Ltd., C1033), H_2_O_2_ (Sinopharm Chemical Reagent Co., Ltd., 1001218), Ethanol hydrochloride rapid differentiation solution (Shanghai Biyuntian, C0163S), Sudan B dye (Beijing Suolebao, G1140), red phosphorus (99.99%, Aladdin), tin (AR, Aladdin), tin tetraiodide (AR, Aladdin), *N*-methylpyrrolidone (AR, Aladdin), APS (ST005), RIPA (P0013B), PMSF (ST506), TEMED (ST728) (Beyotime, China), 30% Acr/Bic (BL513A), Tris-Base (BS083), Prestained Protein Marker II (10 to 200 kDa) (G2058-250UL) (Servicebio China), GAPDH Loading Control Antibody (MA5-15738) (Thermo Fisher Scientific, USA), Goat Anti-Rabbit IgG H&L/HRP(bs-0295G-HRP), Goat Anti-Mouse IgG H&L/HRP (bs-0296G-HRP) (Bioss, China), Drp1 Antibody (D6C7), OPA1 Antibody (D6U6N), Sirt3 Antibody (C73E3), Bax Antibody (#2772), Bcl-2 Antibody (D17C4), YME1L1 Antibody (66551-1-Ig), SIRT3 Recombinant monoclonal antibody (Proteintech, 82210-1-RR, China), and YME1L1 Polyclonal antibody (Proteintech, 11510-1-AP, China).

### Experimental equipment

The following equipment were used in this study: Medical low-temperature storage box DW-86L338J (Qingdao Haier Biomedical Co., Ltd.), incubator and water bath BWS-5 (Shanghai Yiheng Scientific Instrument Co., Ltd.), digital display constant temperature water bath HH-1G (Shanghai Xiniu Laibo Instrument Co., Ltd.), fully automatic snowflake ice maker IMS-30 (Changshu Xueke Electric Co., Ltd.), liquid nitrogen bioculture container YDS-65-216-F (5.0) (Qingdao Haier Biomedical Co., Ltd.), laboratory ultrapure water system UPF-40L-RO (Shanghai Chenxi Instrument Co., Ltd.), CFX96 Touch 1855195 real-time quantitative PCR system and Western blotting system (model: Criterion electrophoresis tank, Trans-Blot transfer tank) (Bio-Rad Laboratories, USA), Orbital Shaker OS-40Pro (JOANLAB Group Safety Instrument Co., Ltd.), Multiskan FC microplate reader 357-714018 (Thermo Fisher Scientific, USA), JP-K6000 chemiluminescence analyzer (Shanghai Jiapeng Technology Co., Ltd.), high-speed bench centrifuge H1850 (Hunan Xiangyi Laboratory Instrument Development Co., Ltd.), CO_2_ incubator CI-150C (Suzhou Jiemai Electronics Co., Ltd.), optical microscope (ZEISS, Germany), super clean workbench SW-CJ-1D (Suzhou Boleier Purification Equipment Co., Ltd.), Keyence-BZ-H4XD Fluorescence Microscope (Japan Keyence Corporation), Attune NxT Flow Cytometer (Thermo Fisher Scientific, USA), tissue dehydrator (Shanghai Leica Instrument Co., Ltd., ASP300), fully automated embedder (Shanghai Leica Instrument Co., Ltd., EG1140H), paraffin sliding glass cutter (Shanghai Leica Instrument Co., Ltd., RM2235), slide spreader (Shanghai Leica Instrument Co., Ltd., HI1210), slide oven (Shanghai Leica Instrument Co., Ltd., HI1220), incubator (Shanghai Foma Laboratory Equipment Co., Ltd., DGX-9003B), microwave oven (Galanz Microwave Oven Appliance Co., Ltd., P70D20P-TF), freeze–thaw sliding glass cutter (Shanghai Leica Instrument Co., Ltd., CM1950), and optical microscope (Olympus, Japan, BX51).

### Synthesis steps of black phosphorus crystals

In this study, we employed the classical chemical vapor deposition method to prepare black phosphorus materials. The specific amounts and experimental procedures are as follows: red phosphorus (500 mg), tin (40 mg), and tin tetrachloride (20 mg) were mixed and separately loaded into a quartz vacuum tube with a length of 300 mm as reaction substrates. The glass tube was then horizontally placed inside a muffle furnace. The specific reaction conditions are as follows: the muffle furnace was heated at a rate of 1.35 °C/min to 750 °C, then maintained at 750 °C for 5 h. Subsequently, it was cooled at a rate of 0.33 °C/min to 500 °C and held at this temperature for 3 h. Finally, it was slowly cooled to 150 °C over 8 h. This reaction condition is a process of programmed heating and cooling. During the slow cooling process, a series of chemical or physical changes occurred in the quartz vacuum tube, and gaseous phosphorus vapor slowly deposited at the bottom of the quartz tube to form highly ordered black crystals. After cooling to room temperature, the obtained black crystals are black phosphorus crystals. Immediately afterward, the quartz tube was placed in a glove box, where the black crystalline material was crushed with external force, collected, and placed in a sealed test tube for convenient use. (The BPNSs were collected after grinding and stored in brown glass bottles under argon protection [O₂ <0.1 ppm, H₂O <0.1 ppm] within a glove box. The samples were kept in a 4 °C light-proof refrigerator with a shelf life not exceeding 4 weeks.)

### Exfoliation of BPNSs

The black phosphorus crystals prepared by the above steps are relatively thick and not conducive to subsequent experiments. Based on this, we adopted a liquid-phase ultrasonic exfoliation method to prepare BPNSs. The specific experimental steps are as follows: 100 mg of the above sample was weighed, ground into fine powder with an agate mortar, and dispersed in 250 ml of N-methylpyrrolidone (NMP). The mixture was sonicated for half an hour to ensure complete dispersion, and then placed in an ultrasonic homogenizer for liquid-phase exfoliation at a power of 650 W for 48 h (−4 °C). After the ultrasonic exfoliation was complete, the resulting solution was subjected to gradient centrifugation. Specifically, the solution was centrifuged at 5,000 rpm for 10 min to remove the unexfoliated, thicker black phosphorus crystals at the bottom; the supernatant was then centrifuged at 8,000 rpm for 10 min to separate the relatively thicker black phosphorus flakes at the bottom; subsequently, the supernatant was centrifuged again at 12,000 rpm for 1 h, and the precipitate obtained was the BPNSs we aimed to prepare.

### Synthesis steps of BPNSs@CORT@Raw264.7@(KKEEE)_3_K

Disperse BPNSs in PBS (pH 7.4), add EDC/NHS (molar ratio 1:1.5) to activate carboxyl groups, and react at room temperature for 2 h. Add CORT (1 mg/ml, dissolved in DMSO) and couple it to the surface of BPNSs via amide bonds (stir in the dark at 4 °C for 12 h), then remove free CORT by ultrafiltration centrifugation (100-kDa cutoff). Culture Raw264.7 macrophages until they reach the logarithmic growth phase, lyse them hypotonically (1 × 10^8^ cells/ml, 10 mM Tris-HCl, pH 7.5), and perform differential centrifugation (500 *g* to remove nuclei, 10,000 *g* to collect membrane fractions). Mix BPNSs@CORT with macrophage membranes (mass ratio 1:2; the membrane protein concentration was determined using the bicinchoninic acid (BCA) method, with protein mass serving as the quantitative standard for “membrane mass”. The mass ratio of BPNSs@CORT to macrophage membrane [1:2] essentially represents the ratio of BPNSs to membrane protein mass) and extrude through a liposome extruder (200 nm pore size) 10 times to form BPNSs@CORT@Raw264.7. React the (KKEEE)₃K peptide segment (with a terminal thiol group) with maleimide groups on the surface of BPNSs@CORT@Raw264.7 (premodified via SMCC linker). The BPNSs@CORT@Raw264.7 samples were first washed with 0.1% sodium dodecyl sulfate (SDS) and resuspended in phosphate buffered saline (PBS), followed by the addition of SMCC (final concentration 1 mM). The mixture was stirred at room temperature for 2 h before ultrafiltration to remove excess SMCC, ultimately yielding maleimide-activated nanoparticles. These nanoparticles were then reacted with thiol-containing (KKEEE)₃K peptide (10 μM) at pH 7.4 and 4 °C for 6 h. Remove free peptide segments by ultrafiltration centrifugation, and determine the modification efficiency using the BCA method (target: 5 to 10 peptides per nanoparticle).

### Morphology and structural characterization

The morphology was characterized by TEM, while thickness and layer count were determined using atomic force microscopy (AFM). Potential measurements were performed with a Nano-ZS nanoparticle analyzer, configured with a helium–neon laser (wavelength λ = 633 nm) at 90° incidence angle and 25.0 ± 0.1 °C measurement temperature. X-ray diffraction (XRD) was employed to determine the crystalline structure of black phosphorus, with XPS used to analyze its chemical composition and bonding modes of oxygen-containing elements with phosphorus. Structural characterization was further confirmed by Raman spectroscopy.

### Stability testing

Disperse BPNSs@CORT in deionized water (DW), DMEM, PBS, and 50% human serum (final concentration, 0.1 mg/ml) respectively, and sonicate for 5 min (power 100 W). The nanoparticles were characterized by dynamic light scattering (DLS) for detection of hydrodynamic diameter size and polydispersity index (PDI). Take an appropriate amount of BPNSs with the same concentration and place them in the same glass container. Take photos of them in the same time point with a mobile phone for 1 week to observe their degradation.

### Hemolysis experiment

Take 1 ml of anticoagulated (EDTA) whole blood, add 9 ml of PBS, centrifuge at 1,500 rpm for 10 min, discard the supernatant, and repeat the washing process 3 times. Prepare a 2% (v/v) red blood cell suspension using PBS. Add 100 μl of red blood cell suspension and 900 μl of the sample to each tube, and gently mix. The samples were incubated in a 37 °C water bath for 4 h, followed by centrifugation at 3,000 rpm for 10 min. Take 200 μl of the supernatant and measure the absorbance at 541 nm (OD value).

### Ultraviolet spectrophotometer detection

A certain amount of BPNSs with a certain concentration was uniformly dispersed in distilled water and exposed to air. Their ultraviolet (UV)–visible spectra were measured and UV absorption peaks were observed during the same period.

### Cell culture

Renal Cell Carcinoma HK-2 was obtained from the American Type Culture Collection in the United States. These cells were cultured in a 37 °C incubator with 95% relative humidity and 5% CO_2_, using DMEM medium supplemented with 10% fetal bovine serum, 100 units/ml penicillin, and 50 units/ml streptomycin. All experiments utilized cells in the logarithmic growth phase.

### Si-SIRT3

The coding sequence of the SIRT3 gene was obtained from NCBI, and SIRT3 siRNA was constructed based on the SIRT3 gene sequence as follows:

**Table:** 

SIRT3 siRNA-1
5′-AAAAGGGCUUGGGGUUGUGAA-3′ 3′-CACAACCCCAAGCCCUUUUUC-5′

**Table:** 

SIRT3 siRNA-2
5′-UCAACCAGCUUUGAGGCAGGG-3′ 3′-CUGCCUCAAAGCUGGUUGAAG-5′

**Table:** 

SIRT3 siRNA-3
5′-AAAGGCUCCACCUCCAGGGAG-3′ 3′-CCCUGGAGGUGGAGCCUUUUG-5′

**Table:** 

si-NC (general negative control for gene companies)
5′-UUCUCCGAACGUGUCACGUTT-3′ 5′-ACGUGACACGUUCGGAGAATT-3′

Cell transfection: Add 2 μl of 20 μM siRNA double-stranded DNA to 200 μl of sterile culture medium, and gently vortex for 5 cycles using a pipette. Add 10 μl of RNAFit (RNA-specific transfection reagent-RNAFit, HB-RF-1000, Hanheng Biotech) to the aforementioned 200-μl mixture and immediately vortex for 10 s to ensure mixing. Incubate at room temperature for 10 min to allow the formation of transfection complexes between the double-stranded siRNA and RNAFit, with the incubation time not exceeding 30 min. Simultaneously, aspirate the original culture medium and replace it with 1.8 ml of preheated fresh complete serum-containing medium. Add the incubated transfection complex to the transfected wells after medium replacement, and gently shake the cell plate in a straight line to mix thoroughly. The final volume of culture medium per well is 2 ml, with a siRNA final concentration of 20 nM. After transfection, incubate the cells in an incubator for 48 h before detection.

### CCK-8 detects cell proliferation

Logarithmic growth phase cell models were seeded at 2,000 cells per well in 96-well plates and incubated at 37 °C with 5% CO_2_ for 6 h. After the cells adhered, different concentrations of drugs were added according to experimental grouping, with 6 replicate wells per group. Cells in each group were incubated for 24 h, and 10 μl of CCK-8 solution was added to each well 2 h before the end of incubation. After incubation, an enzyme was used to measure OD_450_ (optical density at 450 nm).

### ELISA for measuring cytokine expression levels in cells

Serum levels of C-reactive protein (CRP), tumor necrosis factor-alpha (TNF-α), interleukin-6 (IL-6), and NF-κB were measured following the manufacturer’s protocols: Process the experimental cells: Digest with trypsin, wash with PBS, collect into centrifuge tubes, centrifuge at 1,000 rpm for 5 min, then resuspend in corresponding cell culture medium to prepare a cell suspension. Inoculate 5 × 10^4^ cells per well into 96-well plates, setting 3 to 5 duplicate wells. Incubate at 37 °C for 24 h. Standard dilution: Dilute standards in 96-well plates according to ELISA kit instructions. Sample addition: Accurately add 50 μl of standard solution to the plate and 50 μl of test sample to each well. Add samples to the bottom of the plate, avoiding contact with the wall, and gently shake to mix. Incubation: Seal with capping film and incubate at 37 °C for 30 min. Solution preparation: Dilute 30-fold concentrated washing buffer with distilled water to prepare buffer. Washing: Carefully remove capping film, discard liquid, shake off excess, add buffer to each well, let stand for 30 s, repeat 5 times, and dry. Enzyme addition: Add 50 μl of enzyme solution per well (excluding blank wells). Incubation: Seal with capping film and incubate at 37 °C for 30 min. Washing: Carefully remove capping film, discard liquid, shake off excess, add buffer to each well, let stand for 30 s, repeat 5 times, and dry. Color development: First add 50 μl of chromogen A, then 50 μl of chromogen B, mix gently, and incubate at 37 °C in the dark for 15 min. Termination: Add 50 μl of termination solution to each well and terminate the reaction (the blue color turns yellow at this time). Measure the absorbance (OD value) of each well at 450 nm wavelength in sequence.

### EdU detects cell proliferation

Log-phase cells were seeded at 1 × 10^6^ cells per well in a 6-well plate and treated according to the designated groups. Prepare a 2× 5-ethynyl-2ʹ-deoxyuridine (EdU )working solution: Since the EdU solution is added to the plate in equal volume to the culture medium, it should be prepared at 2× dilution. Add an equal volume of 2× EdU working solution (20 μM) preheated at 37 °C to the 6-well plate to achieve a final EdU concentration of 1×. For example, if the target concentration is 10 μM and the original medium volume in the plate is 1 ml, add 1ml of 2× EdU working solution (20 μM) to the plate. Incubate the cells for 2 h. After EdU labeling, remove the culture medium and add 1 ml of fixative. Incubate at room temperature for 15 min. After removing the fixative, wash the cells with 1 ml of washing solution per well for 3 to 5 min each time. Remove the washing solution and add 1 ml of permeabilization buffer (PBS containing 0.3% Triton X-100) per well, then incubate at room temperature for 15 min. Remove the permeabilization solution and wash the cells twice with 1 ml of washing solution per well for 5 min each time.

### MMP level

For a 6-well plate, prepare 1 ml of JC-1 staining working solution per well by diluting 30 μl OF JC-1 (200× stock) with 4.8 ml OF ultrapure water. Vigorously vortex to fully dissolve and mix the JC-1. Add 1.2 ml OF JC-1 staining buffer (5× stock) and mix thoroughly to obtain the final working solution. Remove cells from the 6-well plate, aspirate the culture medium, and wash the cells once with PBS. Add 1 ml of cell culture medium containing serum and phenol red. Add the pre-prepared 1 ml of JC-1 staining working solution and mix thoroughly. Incubate at 37 °C for 20 min in a cell incubator. During incubation, prepare 1× JC-1 staining buffer by mixing 3 ml of JC-1 staining buffer (5× stock) with 12 ml of distilled water, and store it in an ice bath. After incubation, aspirate the supernatant and wash each well twice with 1 ml of 1× JC-1 staining buffer. Finally, add 2 ml of cell culture medium and observe under a fluorescence microscope or laser confocal microscope.

### DCFH-DA probe detects ROS in cells

Log-phase cells were digested with trypsin and inoculated into 2-cm cell culture dishes. When the cell density reached approximately 70%, the cells were treated with the drug for 2 h, followed by the addition of 2ʹ,7ʹ-dichlorodihydrofluorescein diacetate (DCFH-DA) fluorescent probe for 30 min. Fluorescence signals from the DCFH-DA probe were collected at different time points.

### Flow cytometry was used to detect apoptosis in each group

Log-phase cells (1 × 10^6^ per well) were plated in 6-well plates. After adhesion, cells were subjected to starvation treatment for 12 h, followed by group-specific treatments. The cells were incubated at 37 °C in a 5% CO₂ incubator for 24 h. A suspension was prepared by resuspending cells in appropriate medium, centrifuged at 1,000 rpm for 5 min, and the supernatant was discarded. After resuspending the cells in 1 ml of PBS, centrifuge at 1,000 rpm for 5 min and discard the supernatant. Repeat this step once. Add the pre-prepared 1× Annexin V Binding Solution to prepare a cell suspension with a final concentration of 1 × 10^6^ cells/ml. Transfer 100 μl of the cell suspension from step 3 to a new tube. Add 5 μl of Annexin V, FITC conjugate to the cell suspension, followed by 5 μl of PI solution. Incubate in room temperature and avoid light for 15 min. Add 400 μl of 1× Annexin V Binding Solution and perform detection on the instrument within 1 h.

### Immunofluorescence

Cells in the logarithmic growth phase were seeded at 1 × 10^6^ cells per well in a 6-well plate. After adhesion, the cells were subjected to starvation treatment for 12 h and then processed according to the designated groups. Remove the 6-well plate and discard the supernatant. Gently wash it 3 times with PBS, then add 1 ml of 4% paraformaldehyde. Incubate the cells on a shaker at 50 rpm for 20 min. Discard the supernatant, gently wash 3 times with PBS, then add 1 ml of 0.25% Triton X-100 and incubate on a shaker (50 rpm) for 20 min. Gently rinse 3 times with PBS, then add 1 ml of goat serum and incubate for 30 min. Remove the blocking solution, add 1 ml of Bcl-2 and Bax primary antibodies (1:200), and incubate at 4 °C overnight. Wash the primary antibody 3 times with TBST (5 min each time). Add 1 ml of secondary antibody (1:200) and incubate at room temperature in the shaker (50 rpm) for 1 hour. Discard the secondary antibody and wash 3 times with TBST for 5 min each. After 5 min of DAPI staining, remove the DAPI and wash 3 times with TBST for 5 min each. Fix the slides with Fluoroshield containing 4′,6-diaminofluorophenylindole (DAPI) and capture images under a fluorescence microscope. Analyze Bcl-2 and Bax fluorescence intensity using ImageJ software (National Institutes of Health, NIH) (Table S1).

### Immunofluorescence colocalization

Cells were seeded in confocal culture dishes and, after corresponding treatments, transferred to serum-free medium containing prewarmed mitochondrial-specific probes (MitoTracker Deep Red FM, working concentration 50 nM) and incubated for 30 min in a 37 °C, 5% CO_2_ incubator. The probes selectively and stably labeled the mitochondrial network of live cells. After incubation, cells were gently washed 3 times with prewarmed PBS to remove unbound probes. Subsequently, standard immunofluorescence (IF) procedures were performed: 4% polyformaldehyde fixation, 0.25% Triton X-100 permeabilization, goat serum blocking, sequential incubation with SIRT3 primary antibody and corresponding fluorescent secondary antibody, followed by YME1L1 primary antibody and corresponding fluorescent secondary antibody, and final nuclear restaining with DAPI. After slide mounting, multichannel image acquisition was conducted using a laser confocal microscope.

### Transmission electron microscopy

Centrifuge to collect cells (about half the size of a mung bean), fix with 2.5% glutaraldehyde for at least 6 h, then store in a 4 °C refrigerator. Treat with 0.1 MPB for 10 min, 4 consecutive times; followed by 0.2 ml of fixative for 1 h; 0.1 MPB for 10 min, 2 times; ultrapure water for 10 min, 2 times; 50% ethanol for 10 min; 70% ethanol for 10 min; 90% ethanol for 10 min; 90% ethanol:90% acetone (1:1) for 10 min; 90% acetone for 10 min. 100% acetone (left side of fume hood) for 8 min per session (3 sessions total): acetone:resin (1:1) (0.5 ml) for 1 h, acetone:resin (1:2) (0.5 ml) for 1 h (no cell lysis), acetone:resin (1:3) (0.5 ml) overnight (no cell lysis). Replace the resin with 0.4 ml every 3 h on the second day, then polymerize in an oven at 60 °C for 48 h. Lock the fridge, clean up, check safety, turn off the lights, close the door, and take a photo.

### RT-PCR

Log-phase cells (1 × 10^6^ per well) were plated in 6-well plates. After adhesion, cells were subjected to starvation treatment for 12 h, followed by group-specific treatments. The cultures were incubated at 37 °C with 5% CO₂ for 24 h. The plates were then removed, supernatant was discarded, and 500 μl of Buffer RL was added to lyse the cells. The lysed samples were transferred to FastPure gDNA-Filer Columns III and centrifuged at 12,000 rpm for 30 s. Discard Column III, collect the filtrate in the collection tube, add 0.5 times the filtrate volume of anhydrous ethanol, shake vigorously, transfer the entire mixture to FastPure RNA Column III, centrifuge at 12,000 rpm for 30 s, and discard the filtrate. Add 700 μl of Buffer RW1 to FastPure RNA Columns III, centrifuge at 12,000 rpm for 30 s, then discard the filtrate. Add 700 μl of Buffer RW2 (containing anhydrous ethanol) to FastPure RNA Columns III, centrifuge at 12,000 rpm for 30 s, and discard the filtrate. Add 500 μl of Buffer RW2 (containing anhydrous ethanol) to FastPure RNA Columns III, then centrifuge at 12,000 rpm for 2 min. Carefully remove the adsorption column from the collection tube to avoid contamination by the filtrate. Centrifuge at 12,000 rpm for 1 min with the column empty to prevent ethanol contamination. Carefully transfer the adsorption column to a new RNase-free Collection Tube. Add 30 μl of RNase-free ddH_2_O dropwise to the center of the column, let it stand at room temperature for 3 min, then centrifuge at 12,000 rpm for 1 min to elute RNA. The eluate can be washed and centrifuged again for a second elution. Use the Nano 600 to measure RNA concentration and purity. For cDNA synthesis, prepare a 20-μl reaction system: Add 500 ng RNA, 2 μl of 5×gDNA Wiper Mix, and RNase-free water to a 10 μl of volume in a 200-μl Eppendorf tube (enzyme-free). Perform a 2-min 42 °C reaction in a standard PCR machine. Then, add 2 μl of 10× RT Mix, 2 μl of Hiscript III Enzyme Mix, 1 μl of Oligo(dT)20VN, 1 μl of Random Hexamers, and 4 μl of RNase-free water to the mixture. Mix thoroughly and incubate at 37 °C for 15 min, followed by a 5-s 85 °C denaturation step to obtain cDNA, which should be stored at −20 °C. In a 20-μl reaction system, add the following components to a 100-μl Eppendorf tube excluding enzymes: 5 μl of 5-fold diluted cDNA, 4.2 μl of nuclease-free water, 10 μl of 2× Taq Pro Universal SYBP qPCR Master Mix, 0.4 μl of forward primer (10 μM), and 0.4 μl of reverse primer (10 μM). Transfer the premixed reagents into an 8-well plate, seal it, and load it into the CFX96 Touch 1855195 real-time fluorescent quantitative PCR instrument. Set the reaction parameters and perform amplification. Results are calculated using the 2^−ΔΔCt^ method.

### Western blot

Log-phase cells (1 × 10^6^ per well) were plated in 6-well plates. After adhesion, cells were subjected to starvation treatment for 12 h, followed by group-specific treatments. The cultures were incubated at 37 °C with 5% CO₂ for 24 h. The plates were removed, supernatants were discarded, and cells washed twice with precooled PBS. Each well received 0.5 ml of RIPA lysis buffer containing PMSF. After complete lysis, the samples were centrifuged at 4 °C for 5 min at 12,000 *g*. The supernatant was immediately transferred to precooled EP tubes, yielding cellular protein extracts. These were then aliquoted and stored at −80 °C for subsequent use. Prepare BCA working solution by mixing 50 volumes of BCA Reagent A with 1 volume of BCA Reagent B (50:1) based on sample quantity, and thoroughly mix. Fully dissolve the protein standard by adding 10 μl of it to PBS to achieve a final concentration of 0.5 mg/ml. Add the standard solutions (0, 1, 2, 4, 8, 12, 16, and 20 μl) to the standard wells in the 96-well plate, then top up with the standard dilution buffer to reach 20 μl. Add appropriate volumes of sample to the sample wells. If the sample volume is less than 20 μl, supplement with standard dilution buffer to reach 20 μl. Add 200 μl of BCA working solution to each well and incubate at 37 °C for 30 min. Measure absorbance at 562 nm for each well, plot a standard curve, and calculate protein concentration (in mg/ml) using the formula. Finally, add 5× Loading Buffer to achieve a final concentration of 1×, then incubate in boiling water bath for 10 min. The sample is ready for storage at −20 °C. Prepare a 12% separating gel and 5% running gel for SDS-PAGE gel loading. Add a measured amount of precooled 1× electrophoresis buffer to the wells, followed by the previously extracted 20 μg of total intracellular protein and 6 μl of prestained protein marker. Run at 80 V for approximately 30 min. Once the sample enters the separating gel, adjust the voltage to 120 V and continue electrophoresis. Stop when the target band reaches the prestained protein marker position. Cut polyvinylidene fluoride (PVDF) membrane according to gel size, activate in methanol for 1 min, then transfer to transfer buffer. Place filter paper in the buffer for 15 min. Create a “sandwich” transfer setup (gel on negative electrode side, membrane on positive electrode side) to ensure bubble-free electrophoresis. After transfer, wash membrane with TBST for 2 min. At room temperature, block membrane on a shaker for 30 min with blocking solution, then incubate primary antibody overnight at 4 °C. Wash membrane 3 times with TBST (10 min each). Add secondary antibody and incubate at room temperature for 2 h. Wash membrane 3 times with TBST (10 min each). Mix equal volumes of chemiluminescent reagent A and B, then position the PVDF membrane with its protein side down to ensure full contact with the mixture. After 3 min, analyze the membrane using a JP-K6000 chemiluminescence imaging system. Protein expression levels were quantified by analyzing optical density values with ImageJ software, with relative expression calculated as the target protein’s grayscale value divided by the internal reference protein’s grayscale value. The antibody dilution factors are as follows (Table S2).

### Co-immunoprecipitation

After overnight antibody incubation, add 5 μl of Protein A and 5 μl of Protein G, and gently mix at 4 °C for 1 to 3 h or overnight. Centrifuge at 12,000 *g* for 1 min and retain the pellet; wash the pellet with 0.5 ml of 1× Wash buffer, centrifuge at 12,000 *g* for 1 min, and retain the pellet (repeat this step 5 times). Resuspend the pellet in 30 μl of 1× SDS-PAGE loading buffer, vortex, then briefly centrifuge for 30 s to detach beads adhered to the tube wall and allow liquid to settle to the bottom. Place the sample in a 100 °C water bath for 5 min, then perform a 1-min instantaneous centrifugation at 14,000 *g*. Collect the supernatant to obtain the Co-IP sample. Samples not immediately used can be stored at −20 °C. The samples with total protein extracted from the third gel block were subjected to electrophoresis. After adding an appropriate amount of precooled 1× electrophoresis buffer, the samples were loaded into the lanes. A 30-min electrophoresis at 80 V was performed. Once the samples entered the separating gel, the voltage was adjusted to 120 V for continued electrophoresis. When the target bands reached the designated positions (referencing the prestained protein Marker), electrophoresis was terminated. PVDF membranes were cut according to gel size, activated in methanol for 1 min, then immersed in transfer buffer. Filter paper was placed in the buffer for 15 min. Following the principle of (positive electrode) filter paper ≥ PVDF membrane ≥ gel ≥ filter paper (negative electrode), a “sandwich” transfer setup was prepared. After removing air bubbles, constant current transfer (200 mA) was initiated. The process was completed with 3 TBST washes. After dipping the membrane in TBST solution from bottom to top, transfer it to a plate containing blocking solution (5% BSA or 5% skim milk TBST). Incubate at room temperature for 2 h to block the immunoglobulin-binding sites on the PVDF membrane. Wash the membrane 3 times with TBST and rinse off any residual liquid. Incubate at room temperature for 2 h. Add secondary antibody and incubate for 4 h. Wash the membrane 3 times with TBST buffer (10 min each time). Mix equal volumes of chemiluminescent reagent A and B, and ensure the membrane is fully exposed to the mixture with the protein side down. Detect using the JP-K6000 chemiluminescence imaging system after 5 min. Analyze the optical density values with ImageJ software to determine protein expression levels. The relative protein expression is calculated as the grayscale value of the target protein divided by the grayscale value of the internal reference protein.

### Animal culture

C57BL/6 mice were housed in cages individually, with bedding changed regularly. The mice had free access to water and food, 6 per cage. The temperature was maintained at 23 ± 1 °C, and the room had a 12-h light–dark cycle. Five days before the experiment, the mice were allowed to acclimate to the living environment.

### SA-AKI

All mice were housed in our institute’s animal center with a 24-h circadian rhythm and free access to food and water. They underwent 2 days of routine housing before surgery to minimize environmental stress. The procedure employed a 3.5% to 4.5% oxygen–isoflurane mixture at 21 ml/min, with anesthesia maintained until the mice showed pain-free withdrawal reflexes when footpads were pinched. The abdomen was disinfected twice with 70% alcohol. A 1-cm subxiphoid midline incision was made, and the peritoneum was carefully lifted using forceps to avoid damaging adjacent organs. The peritoneum was incised along the midline to access the abdominal cavity, with meticulous care taken to locate the cecum along the incision without excessive manipulation. Upon identification, the cecum was gently retracted using blunt-tipped forceps or cotton swabs. The ileocecal junction was measured, and a 2.0-gauge suture was ligated at 25% of the cecal length. A 21G needle was inserted through the cecum (2 holes) to aspirate fecal matter, after which the cecum was repositioned. The peritoneum and skin were layered and sutured, followed by resuscitation with 1 ml of warm saline solution.

### Hematoxylin and eosin

After 12 h of fixation, rinse with running water for 1 h, and store in 75% ethanol for a long time. The tissue was successively dehydrated with ethanol solutions of different concentrations (70%, 80%, 90%, 95%, 100%, and 100%) for 40 min at each concentration. The tissue was sequentially soaked in 3 xylene solutions, with each cylinder treated for 1 hour. The tissue was sequentially soaked in 3 paraffin cylinders for 1 h each. Pour liquid paraffin into the mold box, then place the wax-impregnated tissue block flat on the bottom with the cut surface facing downward. Once the paraffin solidifies, remove the embedding frame. After complete cooling and hardening, trim the paraffin block. Retain a moderate amount of paraffin around the tissue for subsequent sectioning. Secure the precooled paraffin block on the paraffin slicer with the cutting surface parallel to the blade. Set the blade angle to 15°, rotate the feed wheel, and adjust the thickness to 4 μm for uniform slices. Hold the brush in your left hand while turning the slicer handle with your right. Gently lift the sliced paraffin block with the brush, then carefully remove it using tweezers. Place the block face-down into the mounting chamber (45 °C water bath). After smoothing the surface, remove the block. Adhesion: Hold one end of the slide vertically in water to attach the sliced block. Use tweezers to assist in positioning it two-thirds of the way along the slide. After cutting and adhering the slices, let them air-dry briefly, then bake for 1 h in a 65 °C slice oven, followed by 2 h in an oven. The paraffin sections underwent a gradient dewaxing process, sequentially treated with Xylene I (10 min), Xylene II (10 min), Xylene III (10 min), Anhydrous Ethanol I (5 min), Anhydrous Ethanol II (5 min), 90% Alcohol (5 min), 80% Alcohol (5 min), 70% Alcohol (5 min), and 50% Alcohol (5 min). Paraffin sections were stained with hematoxylin for 1 min, rinsed with tap water, and subjected to 1% HCl alcohol differentiation for several seconds. After another rinse, they were treated with 1% ammonia solution for 1 min to restore blue coloration. Following this, the sections were washed with running water for a few seconds, then dyed with eosin for several seconds, and rinsed with running water. Finally, the specimens were dried and photographed under a microscope.

### Immunohistochemistry and functional assessments

Antigen retrieval was performed in citrate buffer at 95 °C for 15 min. Sections were incubated overnight at 4 °C with primary antibodies. DAB (Servicebio) was used for visualization. Place melted paraffin into the embedding block. Remove tissues from the dehydration box before solidification, then insert them into the embedding block according to requirements and affix corresponding labels. Cool on a −20 °C freezer platform. After solidification, remove and trim the wax blocks. Place the embedded wax blocks in a 4 °C refrigerator for shaping, with specific time determined by subsequent experiments. Slicing: After 10-h shaping, retrieve from the 4 °C refrigerator and attach to the microtome. First, roughly cut 1-mm-thick wax sections to establish tissue planes, then finely slice into 5-μm sections. Spread prepared sections onto a 45 °C water bath using tweezers. Once flat, transfer to a slide and air-dry before drying in a 37 °C oven. Dehydration and waterization: Pre-dehydrate sections in a 60 °C oven for 20 min. Soak sections in fresh xylene for 5 min 3 times. Soak in anhydrous ethanol for 5 min twice. Sequentially immerse in 95% ethanol (5 min), 85% ethanol (5 min), and 75% ethanol (5 min), followed by PBS rinsing 3 times (5 min each). Antigen retrieval: Microwave 1×EDTA antigen retrieval solution until it boils. Submerge sections and simmer on low heat for 15 min. Allow cooling, rinse 3 times with PBS (5 min each), then dry slides with blotting paper. Intra-endogenous peroxidase block: Place sections in a 3% hydrogen peroxide solution and incubate at room temperature for 10 min to block endogenous peroxidase. Wash with PBS 3 times, 5 min each, then dry thoroughly. Antibody hybridization: Cover tissues with 5% BSA by droplet on sections, blocking at room temperature for 20 min. Remove BSA, add 50 μl of diluted antibody (1:100 dilution) per section, and incubate overnight at 4 °C. After 4 °C incubation, allow sections to warm at room temperature for 45 min; wash with PBS 3 times, 5 min each; remove PBS, add 50 to 100 μl of diluted secondary antibody per section, and incubate at room temperature for 30 min. Wash with PBS 3 times, 5 min each; remove PBS, add 50 to 100 μl of freshly prepared DAB solution per section, and observe color development under microscope. Rinse with distilled water after complete color development to stop reaction. Safranin staining: Stain sections in safranin stain and observe staining time under microscope. Place stained sections in 1% hydrochloric acid alcohol for 1 s, rinse with tap water. Dehydration and mounting: Dehydrate sections sequentially through 75% ethanol (10 min), 85% ethanol (10 min), 95% ethanol (10 min), and absolute ethanol (10 min). Mount sections in xylene for 5 min, repeating 3 times. Mounting and photographing: Mount with neutral resin carefully to avoid bubbles. Observe and photograph under an optical microscope.

### Single-cell RNA-seq bioinformatics analysis

The single-cell nuclear transcriptome data of human kidneys used in this study were sourced from the Gene Expression Omnibus (GEO) public database, with accession number GSE210622. The study included renal tissue samples from 3 patients with severe AKI and 3 non-AKI controls, all of whom were non-COVID-19 cases. Raw data were preprocessed using the R language Seurat package, with expression matrices read from each sample and Seurat objects independently constructed. Preliminary filtering criteria were set as follows: each cell must detect at least 200 genes, and each gene must be expressed in at least 3 cells. Subsequently, Seurat objects from 6 samples were merged into a unified object for subsequent analysis.

### RNA-seq

The RNA sequencing samples included the model group (CLP+PBS) and the treatment group [CLP+BPNSs@CORT@Raw264.7@(KKEEE)₃K]. Total RNA was extracted from renal tissue and mixed in equimolar proportions for sequencing analysis. After extracting total RNA from the sample, eukaryotic mRNA is enriched using magnetic beads conjugated with Oligo(dT) (for prokaryotes, rRNA is removed using a kit before proceeding to the next step). Fragmentation buffer is added to break the mRNA into short fragments. Using the mRNA as a template, the first cDNA strand is synthesized with random hexamers. The second cDNA strand is then synthesized by adding buffer, dNTPs, RNase H, and DNA polymerase I. Subsequently, the double-stranded cDNA undergoes end repair, poly(A) addition, and sequencing adapter ligation. Magnetic beads are used for purification and fragment selection, followed by PCR amplification to obtain the library. After library quality control (QC) is passed, sequencing is performed. The resulting data are termed raw reads or raw data, which undergo QC to determine their suitability for subsequent analysis. Following QC, clean reads are filtered and aligned to the reference sequence. The alignment results are evaluated by statistically analyzing the distribution and coverage of reads on the reference sequence to determine whether the alignment passes the second QC (QC of alignment). If approved, a series of follow-up analyses will be conducted, including gene expression, alternative splicing, prediction of novel transcripts, SNP detection, and gene structure optimization. Based on the gene expression results, differentially expressed genes between samples will be screened. Using these differentially expressed genes, marked enrichment analyses of GO functions and Kyoto Encyclopedia of Genes and Genomes (KEGG) pathways will be performed.

### Statistical analysis

Statistical analysis and figure generation were performed using GraphPad Prism 9 (Version 9.5.1) and ImageJ software. All data were organized and combined using Adobe Illustrator (2023). All data were presented as means ± SD. Two-tailed *t* tests were used for pairwise comparisons, while one-way analysis of variance was employed for multiple comparisons. Significant differences were defined as *P* values <0.05 (marked by *), <0.01 (marked by **), and <0.001 (marked by ***).

## Results

### Public single-cell data reveal mitochondrial dynamic imbalance and Sirt3 down-regulation in renal tubular epithelial cells during AKI

**Fig. 1.**
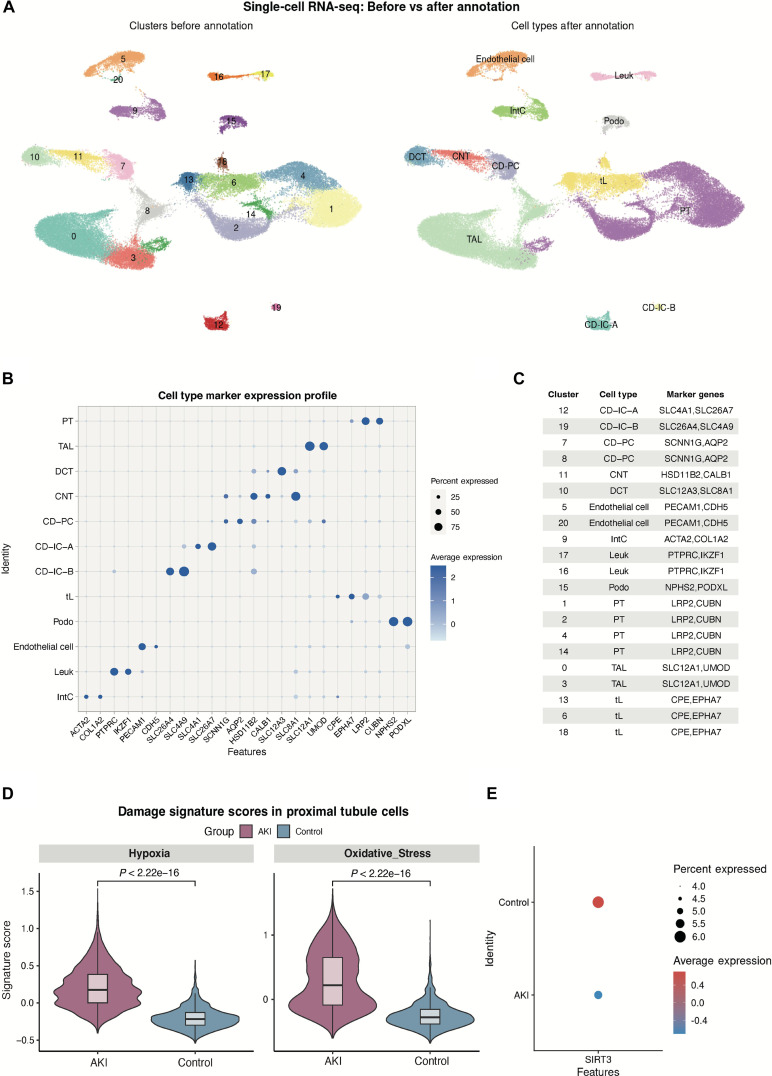
Single-nucleus transcriptomic profiling reveals renal cell heterogeneity and activation of injury-associated pathways in human acute kidney injury (AKI). (A) Uniform Manifold Approximation and Projection (UMAP) visualization of kidney cells from 3 severe AKI patients and 3 non-AKI controls (dataset: GSE210622). The left panel shows the initial clustering, and the right panel shows the annotated major cell types based on canonical marker genes. (B) Dot plot displaying the expression of key marker genes used to define each cell cluster. Dot size represents the percentage of cells expressing the gene, and color intensity indicates the average expression level. PT, proximal tubule; TAL, thick ascending limb; DCT, distal convoluted tubule; CNT, connecting tubule; CD-PC, collecting duct principal cell; CD-IC, collecting duct intercalated cell; EC, endothelial cell; Podo, podocyte; Leuk, leukocyte; IntC, interstitial cell; tL, thin limb. (C) Table summarizing the top marker genes and corresponding cell-type identity for each cluster identified in panel (A). (D) Violin plots comparing the activity scores of 2 injury-associated transcriptional programs—oxidative stress and hypoxia—specifically in proximal tubule (PT) cells between control and AKI groups. The score for each cell was calculated as the average expression of a curated gene set (see Materials and Methods). Statistical significance was determined by Wilcoxon rank-sum test. (E) Dot plot showing the total expression of Sirt3 in control and AKI samples.

We first focused on and analyzed the single-cell sequencing data of AKI. The experimental results, as shown in Fig. 1, reveal that the single-cell transcriptomic atlas untangles renal cellular heterogeneity and the activation of injury-associated pathways in human AKI. Figure 1A demonstrates a distinct distribution difference in the overall transcriptomic profiles of renal cells between AKI patients and the control group. Major cell types, including proximal tubules, thick ascending limbs, distal convoluted tubules, principal cells of the collecting duct, endothelial cells, podocytes, leukocytes, interstitial cells, and thin limbs, were clearly distinguished through marker gene annotation. Figure 1B shows that the cell type-specific marker genes are highly expressed in their corresponding cell clusters, confirming the accuracy of the cell annotation. Figure 1C summarizes the cell types corresponding to each cluster and their primary marker genes, providing a basis for cellular identity in subsequent analyses. Violin plots (Fig. 1D) indicate that, compared to the control group, oxidative stress and hypoxia scores in proximal tubular cells from AKI patients are significantly elevated, suggesting that proximal tubular cells are in a state of severe oxidative damage and hypoxia during AKI. Given the close association between oxidative stress and mitochondrial dysfunction, and considering that Sirt3 is a key deacetylase regulating mitochondrial homeostasis, we hypothesized that the down-regulation of the Sirt3 signaling axis might be a critical link in the aforementioned injury state. The dot plot (Fig. 1E) shows that the expression level of Sirt3 is down-regulated in AKI samples compared to the control group, suggesting that Sirt3 may be involved in the pathological process of AKI.

### Characterization of BPNSs@CORT@Raw264.7@(KKEEE)_3_K structures and morphologies

**Fig. 2.**
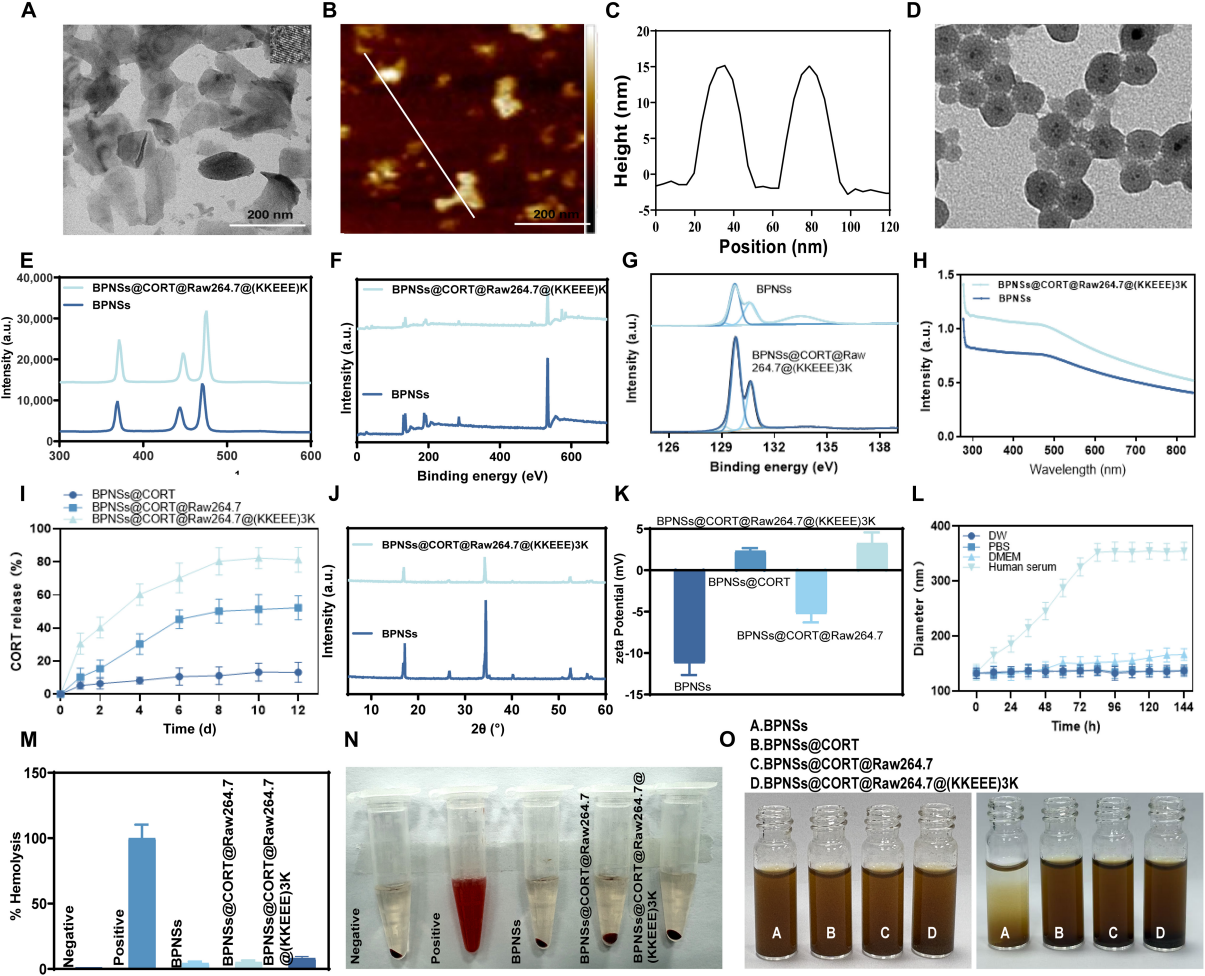
Synthesis and characterization of BPNSs@CORT@Raw264.7@(KKEEE)_3_K. (A) TEM of BPNSs. (B and C) AFM of BPNSs. (D) TEM OF BPNSs@CORT@Raw264.7@(KKEEE)_3_K. (E) Raman of BPNSs and BPNSs@CORT@Raw264.7@(KKEEE)_3_K. (F and G) XPS of BPNSs and BPNSs@CORT@Raw264.7@(KKEEE)_3_K. (H) UV of BPNSs and BPNSs@CORT@Raw264.7@(KKEEE)_3_K. (I) CORT drug release rate. (J) XRD of BPNSs and BPNSs@CORT@Raw264.7@(KKEEE)_3_K. (K) Zeta potential analysis. (L) Stability of BPNSs@CORT@Raw264.7@(KKEEE)_3_K. (M and N) Hemolysis test. (O) Deconstruct image.

To characterize the synthesized BPNSs and the composite BPNSs@CORT@Raw264.7@(KKEEE)₃K, the following instruments were employed for characterization, and the experimental results are shown in Fig. 2. First, the morphology and structural features of BPNSs were examined (Fig. 2A). The prepared BPNSs exhibited a length of approximately 200 nm and a width of about 300 nm. Morphologically, they appeared as irregular polygons, similar to most 2-dimensional materials. Generally, the number of layers, thickness, and transparency of nanosheets are closely related to the exfoliation time. High-resolution TEM was used to observe the lattice fringes of BPNSs. As shown in Fig. 2A, lattice fringes were visible, which could be attributed to the (021) plane of black phosphorus crystals. Subsequently, the thickness of BPNSs was determined using AFM to estimate the number of layers. As shown in Fig. 2B and C, the thickness of the BPNS system ranged from approximately 1.2 to 2.3 nm, indicating that the nanosheets consisted of about 1 to 3 layers. Figure 2D represents the electron microscopy image of BPNSs@CORT after being coated with macrophage membranes. Next, Raman spectroscopy was utilized to characterize the chemical structure. As shown in Fig. 2E, the Raman spectrum of BPNSs exhibited characteristic absorption peaks at 366.5, 433.4, and 457.9 cm^−1^, which can be assigned to one out-of-plane phonon mode (A1g) and 2 in-plane modes (B2g and A2g), respectively. Subsequently, the characteristic absorption peaks of BPNSs@CORT@Raw264.7@(KKEEE)₃K were detected by Raman spectroscopy, revealing 3 blue-shifted peaks similar to those of pristine BP at 368.7, 438.8, and 461.1 cm^−1^. This indicates that the Raman peaks of the two are highly similar but exhibit slight differences. The observed blue shift in the characteristic absorption peaks may be attributed to the binding of CORT. To further elucidate the binding mode of CORT to black phosphorus, XPS was employed to investigate the chemical composition and electronic states of BPNSs and BPNSs@CORT@Raw264.7@(KKEEE)₃K. As shown in Fig. 2F and G, 2 characteristic peaks corresponding to P 2p₃/₂ and P 2p₁/₂ of pristine BP were observed at 129.4 and 131.8 eV, respectively. In Fig. 2F, 2 characteristic peaks of P 2p₃/₂ and P 2p₁/₂ were also detected at 129.8 and 131.3 eV for BPNSs@CORT@Raw264.7@(KKEEE)₃K, indicating the presence of P–P bonds. Additionally, a peak at 134.6 eV was observed for BPNSs@CORT@Raw264.7@(KKEEE)₃K, corresponding to P–O bonds. UV spectroscopy was further used to verify the binding mode, as shown in Fig. 2H. The drug release profile is shown in Fig. 2I, demonstrating that the drug release rate increases progressively over time following encapsulation and targeting by macrophage membranes. Subsequently, XRD analysis was performed to characterize the crystal phases of the 4 materials. As shown in Fig. 2J, distinct characteristic diffraction peaks of black phosphorus were observed at 15.9°, 25.9°, 33.6°, and 52.7°, which were assigned to the (020), (021), (040), and (060) crystal planes of BP, respectively, by comparison with the standard card of black phosphorus. This confirmed the successful synthesis of BP. The crystal structure of BPNSs@CORT@Raw264.7@(KKEEE)₃K was also examined using XRD. As shown in Fig. 2J, characteristic diffraction peaks similar to those of BP were observed at 16.5°, 25.8°, 34.3°, and 52.4°, corresponding to the (020), (021), (040), and (060) crystal planes of black phosphorus, respectively. The XRD results demonstrated that BPNSs@CORT@Raw264.7@(KKEEE)₃K possesses a crystal structure highly similar to that of BP, confirming the successful synthesis of both materials. Moreover, it was shown that the introduction of appropriate amounts of CORT, Raw264.7, and (KKEEE)₃K did not affect the crystal structure of BP.

Furthermore, we conducted quantitative analysis to supplement the encapsulation efficiency (EE) and drug loading capacity (DL) of nanoparticles. Table S3 demonstrates the encapsulation efficiency of the composite nanomaterial BPNSs@CORT@Raw264.7@(KKEEE)₃K, showing a drug encapsulation rate of 86.14% ± 3.60%, indicating excellent encapsulation performance of this composite material. Additionally, we evaluated the drug-loading capacity of the composite nanomaterial. As shown in Table S4, experimental results indicate that the average drug loading capacity of the BPNSs@CORT@Raw264.7@(KKEEE)₃K nanocomposite system is 16.79, confirming that this composite nanomaterial possesses outstanding encapsulation efficiency and drug-loading capability. The release profile of CORT was subsequently investigated. As shown in Fig. 2I, the cumulative release of CORT from BPNSs@CORT alone was less than 20% after 12 days, while that from BPNSs@CORT@Raw264.7 was less than 60%. After targeting with (KKEEE)₃K, the final system achieved a drug release rate of nearly 80%. These results indicate that drug release was significantly improved following macrophage membrane coating and targeting. This difference is primarily attributed to alterations in the physicochemical properties of the nanoparticles themselves rather than biometric recognition. The macrophage membrane is predominantly composed of a phospholipid bilayer and embedded membrane proteins. When encapsulated on the surface of BPNSs@CORT, it forms a hydrophilic, protein-rich “coronal” shell. This shell significantly enhances the dispersion and colloidal stability of the original BPNSs@CORT (which may exhibit hydrophobic surfaces or different charges) in PBS aqueous solution. Improved dispersion results in a larger effective surface area exposed to the release medium, thereby facilitating the diffusion of internal CORT. Secondly, under in vitro conditions of constant temperature oscillation at 37 °C, the membrane structure may undergo localized fluidity changes, form transient micropores or defects, or exhibit increased permeability due to conformational alterations of membrane proteins. Additionally, the lack of cellular energy metabolism environments that maintain membrane integrity in PBS may lead to slow hydration, swelling, or even partial disintegration of membrane vesicles themselves, all of which accelerate the release of CORT encapsulated within or embedded in the membrane. Finally, the (KKEEE)₃K peptide modification peptide segment, rich in negatively charged glutamic acid (E) and positively charged lysine (K), further fine-tunes the hydrophilicity, charge distribution, and interfacial energy of nanoparticle surfaces. This may influence the permeation rates of water molecules and ions into the particle interior or alter the dissociation energy barrier of CORT from carrier materials, thereby modulating release kinetics. Furthermore, Zeta potential measurements were conducted to examine the differences in surface potential between BPNSs and BPNSs@CORT@Raw264.7@(KKEEE)₃K. As shown in Fig. 2K, the Zeta potential of pristine BP was approximately –12 mV, while that of BPNSs@CORT was about +2.4 mV. The potential of BPNSs@CORT@Raw264.7 was –5.2 mV, and the final system BPNSs@CORT@Raw264.7@(KKEEE)₃K exhibited a potential of +3.2 mV. These results suggest that the introduction of CORT, Raw264.7, and (KKEEE)₃K influences the surface potential of BP, indirectly supporting the successful synthesis of BPNSs@CORT@Raw264.7@(KKEEE)₃K. The stability of BPNSs@CORT@Raw264.7@(KKEEE)₃K in different solutions (DW, PBS, DMEM, and human serum) was further investigated. As shown in Fig. 2L, BPNSs@CORT@Raw264.7@(KKEEE)₃K exhibited remarkable stability in DW, PBS, and DMEM. In human serum, the increase in particle size is attributed to the formation of protein crowns through binding with proteins and other components in the serum [43]. Hemolysis rate tests (Fig. 2M and N) confirmed that neither individual components nor the final system induced hemolysis, indicating good biocompatibility. Finally, equal concentrations of nanosheets were placed in identical glass containers, and photographs were taken at the same time point over 1 week to observe degradation. As shown in Fig. 2O, pristine BP gradually degraded over time, with the color becoming increasingly transparent. In contrast, the introduction of CORT, Raw264.7, and (KKEEE)₃K reduced the degradation rate of BPNSs.

### Detection of cellular phenotypes

**Fig. 3.**
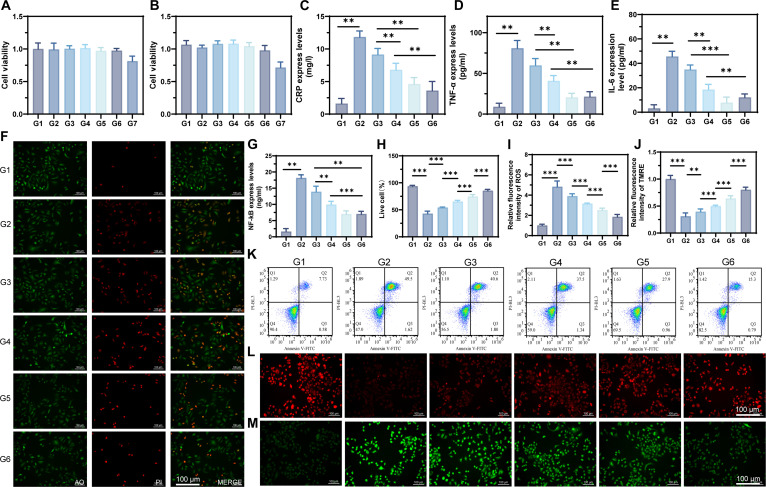
Detection of cellular phenotypes. (A) CCK-8 of BPNSs. (B) CCK-8 of CORT. (C, D, E, and G) ELISA check CRP, TNF-α, IL-6, and NF-kB. (F and H) EdU live staining. (I and M) Fluorescent ROS detection. (J and L) Mitochondrial membrane potential JC-1. (K) Flow cytometric detection of apoptosis. **P < 0.01, ***P < 0.001.

To further validate the therapeutic efficacy of the drug, a series of cellular functional assays were conducted, as shown in Fig. 3. Initially, the concentrations of BPNSs and the CORT agonist corticosterone were screened. The results (Fig. 3A and B) indicated that 4 μg/ml BPNSs and 4 μM CORT were selected for subsequent experiments. The experimental groups were then designated as follows: G1, Control group; G2, LPS group; G3, BPNSs group; G4, BPNSs@CORT group; G5, BPNSs@CORT@Raw264.7 group; and G6, BPNSs@CORT@Raw264.7@(KKEEE)₃K group. First, the expression levels of inflammatory factors, including CRP, TNF-α, IL-6, and NF-κB, were measured using ELISA. As shown in Fig. 3C, D, E, and G, the G6 group exhibited a significantly greater inhibition of inflammation compared to the other groups. Subsequently, cell viability was assessed using the EdU assay. The results (Fig. 3F and H) demonstrated that the G6 group effectively promoted and ameliorated LPS-induced cell death. Although some improvement was observed in the other groups, the extent of recovery was substantially less than that in the G6 group. Apoptosis was further evaluated by flow cytometry. As shown in Fig. 3K, apoptosis was effectively suppressed in the G6 group. Furthermore, cellular oxidative stress was investigated by measuring ROS levels and MMP. The results (Fig. 3L, J, M, and I) revealed that LPS stimulation induced a significant increase in ROS production and a decrease in MMP. However, treatment with G3, G4, G5, and G6 groups led to varying degrees of improvement in these parameters. Collectively, these findings indicate that BPNSs@CORT@Raw264.7@(KKEEE)₃K effectively inhibits LPS-induced apoptosis and demonstrates a strong capacity to counteract oxidative stress.

### Composite nanosystem mediates SIRT3 regulation of apoptosis and mitochondrial function detection in cells

**Fig. 4.**
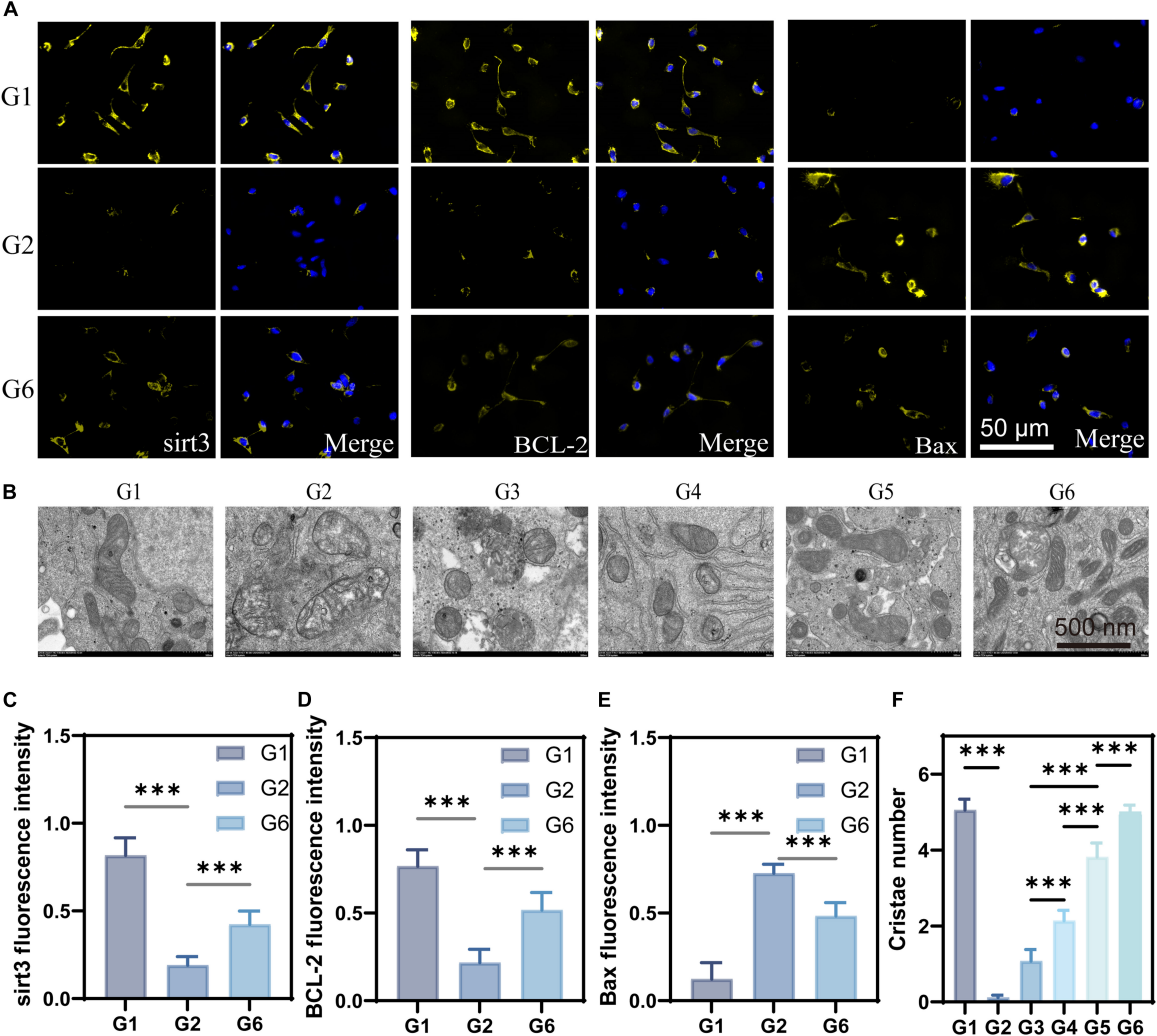
Detection of apoptosis and mitochondrial function. (A, C, D, and E) Immunofluorescence was used to detect the expression of Sirt3, Bax, and BCL-2 in the ABF group. (B and F) Transmission electron microscopy detects mitochondrial damage. ***P < 0.001.

To further validate the anti-apoptotic capacity of the final system drug BPNSs@CORT@Raw264.7@(KKEEE)₃K, IF and TEM were employed to detect apoptosis markers and mitochondrial function. The experimental results are shown in Fig. 4. Firstly, IF was used to detect the apoptosis markers SIRT3, BCL-2, and Bax. As shown in Fig. 4A, C, D, and E, the expression level of SIRT3 was significantly increased in the G6 group, accompanied by increased BCL-2 and decreased Bax levels. These results indicate that BPNSs@CORT@Raw264.7@(KKEEE)₃K effectively inhibits apoptosis. Furthermore, mitochondrial morphology was examined by TEM. Mitochondria, as the energy-producing organelles of the cell, often appear oval or elongated in cross-section. Their structure, from the outside inward, consists of the outer membrane, intermembrane space, inner membrane, and matrix, with the inner membrane folding inward to form cristae. The results, shown in Fig. 4B and F, revealed that LPS treatment induced mitochondrial swelling, shortened and disorganized cristae, and a significant decrease in matrix electron density. However, after treatment with G3, G4, G5, and G6 groups, the mitochondrial morphology was improved and gradually restored, particularly in the G6 group, where the mitochondrial morphology was essentially consistent with that of the normal control group G1. These findings demonstrate that BPNSs@CORT@Raw264.7@(KKEEE)₃K effectively ameliorates LPS-induced mitochondrial dysfunction.

### Detection of mitochondria-related genes and proteins

**Fig. 5.**
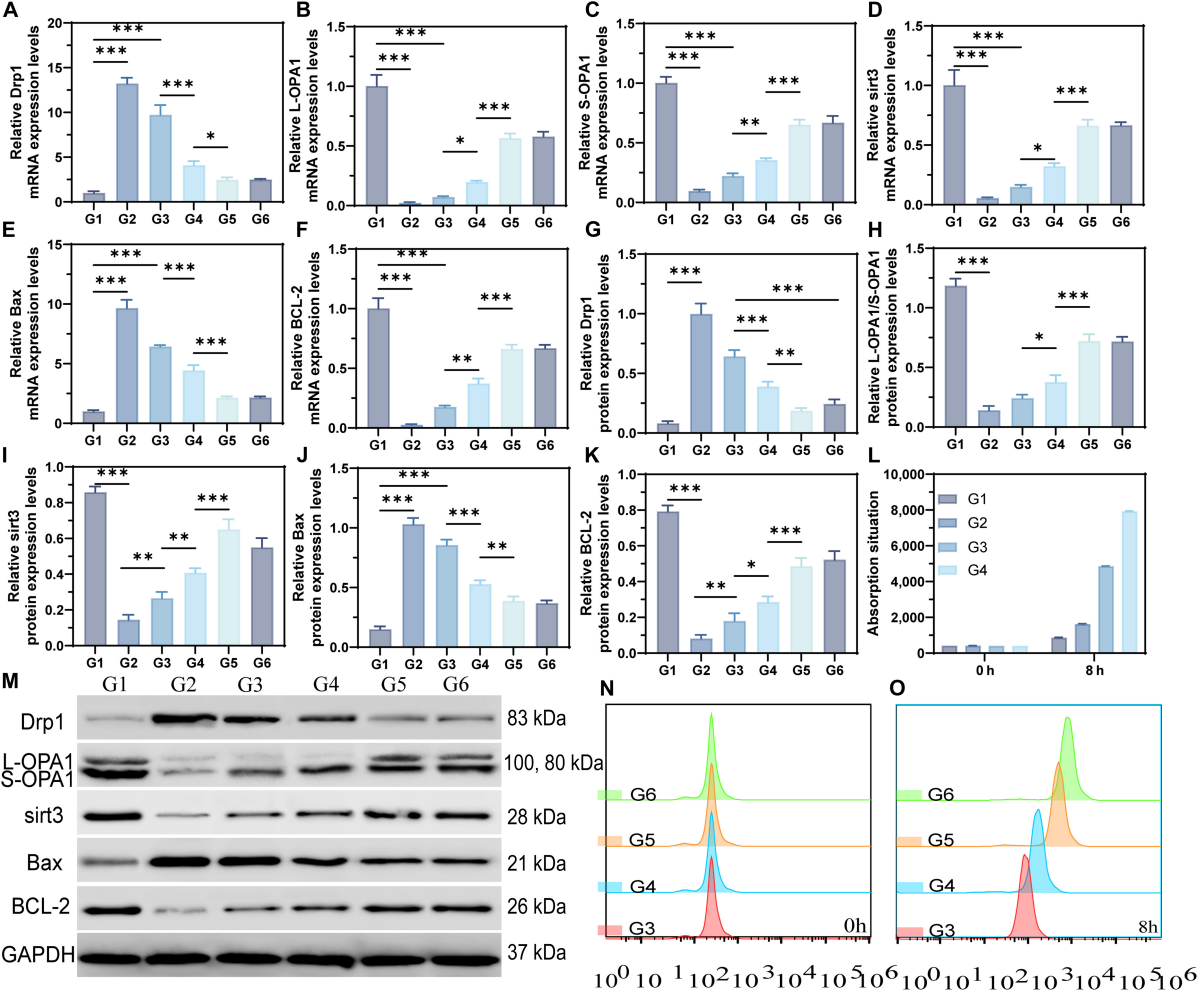
Detection of mitochondria-related genes and proteins. (A to F) RT-PCR detection of Drp1, L-OPA1, S-OPA1, Sirt3, Bax, and BCL-2 expression. (G to K and M) WB detection of Drp1, L-OPA1, S-OPA1, Sirt3, Bax, and BCL-2 expression. (L, N, and O) Flow cytometric detection of cell absorption and uptake. *P < 0.05, **P < 0.01, ***P < 0.001.

SIRT3 is a mitochondrial deacetylase that indirectly influences cell survival and apoptosis by regulating multiple substrates (including antioxidant enzymes, metabolic enzymes, and mitochondrial dynamic proteins). To further validate the impact of the final BPNSs@CORT@Raw264.7@(KKEEE)₃K system drug on mitochondrial function, dynamin-related protein 1 (Drp1), mitochondrial dynamin-like protein OPA1, and apoptosis-related proteins were detected by RT-PCR and Western blot (WB). The experimental results are shown in Fig. 5, with groups designated as follows: G1, Control group; G2, LPS group; G3, BPNSs group; G4, BPNSs@CORT group; G5, BPNSs@CORT@Raw264.7 group; and G6, BPNSs@CORT@Raw264.7@(KKEEE)₃K group. Drp1 is a core regulator of mitochondrial dynamics and plays a critical role in maintaining mitochondrial morphology, function, and cellular homeostasis. OPA1 is a mitochondrial dynamin-like GTPase responsible for mitochondrial morphogenesis, fusion, and energy dynamics. Based on this, RT-PCR was used to detect the mRNA levels of Drp1, L-OPA1, S-OPA1, Sirt3, Bax, and BCL-2. The results, shown in Fig. 5A to F, demonstrated that the G6 final system drug group effectively reduced the levels of Drp1 and Bax, while up-regulating the expression of OPA1, Sirt3, and BCL-2. Further validation at the protein level yielded consistent results, as shown in Fig. 5G to K and M, where the G6 final system drug group similarly reduced Drp1 and Bax protein levels and up-regulated the expression of OPA1, Sirt3, and BCL-2. Additionally, cellular uptake was assessed by flow cytometry. The results, presented in Fig. 5L, N, and O (where N represents cellular uptake at 0 h and O represents uptake after 8 h), indicated that the G6 group, modified with cell membrane coating and conjugated with the targeting peptide (KKEEE)₃K, significantly promoted cellular absorption, thereby achieving homologous targeting.

### Assessment of cellular and mitochondrial function

**Fig. 6.**
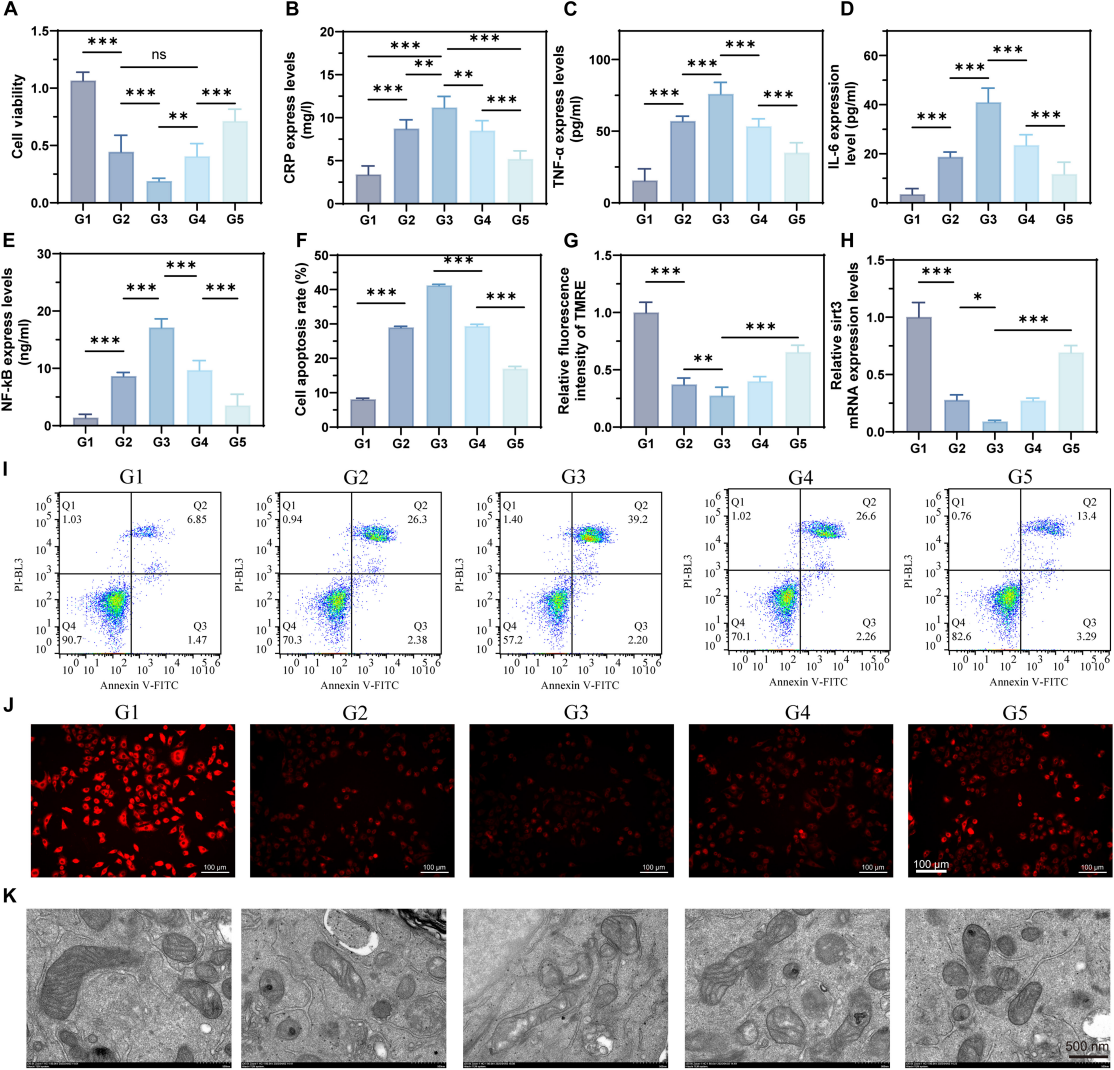
Assessment of cellular and mitochondrial function. (A) CCK-8. (B to E) ELISA detection of CRP, TNF-α, IL-6, and NF-kB expression. (F and I) Flow cytometric detection of apoptosis. (G and J) Mitochondrial membrane potential JC-1. (H) RT-PCR detection of Sirt3 expression. (K) Transmission electron microscopy detects mitochondrial damage. *P < 0.05, **P < 0.01, ***P < 0.001.

To further investigate the mechanism by which BPNSs@CORT@Raw264.7@(KKEEE)₃K treatment improves cell viability, cellular phenotypes were assessed following Sirt3 gene knockdown. The experimental results are shown in Fig. 6, with groups defined as follows: G1, Control; G2, LPS; G3, LPS + si-Sirt3; G4, LPS + si-NC-Sirt3; and G5, LPS + BPNSs@CORT@Raw264.7@(KKEEE)₃K. Initially, cell viability was measured using the CCK-8 assay. As shown in Fig. 6A, Sirt3 knockdown resulted in a further reduction in cell viability, whereas treatment with BPNSs@CORT@Raw264.7@(KKEEE)₃K partially restored it. Subsequently, the levels of inflammatory markers, including CRP, TNF-α, IL-6, and NF-κB, were evaluated using ELISA kits. According to Fig. 6B to E, Sirt3 knockdown led to increased inflammatory levels, which were attenuated following intervention with BPNSs@CORT@Raw264.7@(KKEEE)₃K. Apoptosis was then analyzed by flow cytometry (Fig. 6F and I). Figure 6H demonstrates successful Sirt3 knockdown. Sirt3 knockdown exacerbated apoptosis, while BPNSs@CORT@Raw264.7@(KKEEE)₃K treatment reduced it. MMP was assessed using the JC-1 assay (Fig. 6G and J). Knockdown of Sirt3 further decreased MMP, which was partially restored after BPNSs@CORT@Raw264.7@(KKEEE)₃K administration. Finally, mitochondrial morphology was examined by TEM (Fig. 6K). Sirt3 knockdown induced mitochondrial swelling, shortened and disorganized cristae, and a marked decrease in matrix electron density. In contrast, treatment with BPNSs@CORT@Raw264.7@(KKEEE)₃K (G5) improved mitochondrial morphology, promoting gradual recovery. These results indicate that BPNSs@CORT@Raw264.7@(KKEEE)₃K exerts its therapeutic effects by up-regulating Sirt3 expression, reducing inflammation, and improving mitochondrial function.

### Sirt3 regulates OPA1-mediated mitochondrial fusion by deacetylating YME1L1 in HK-2 cells

**Fig. 7.**
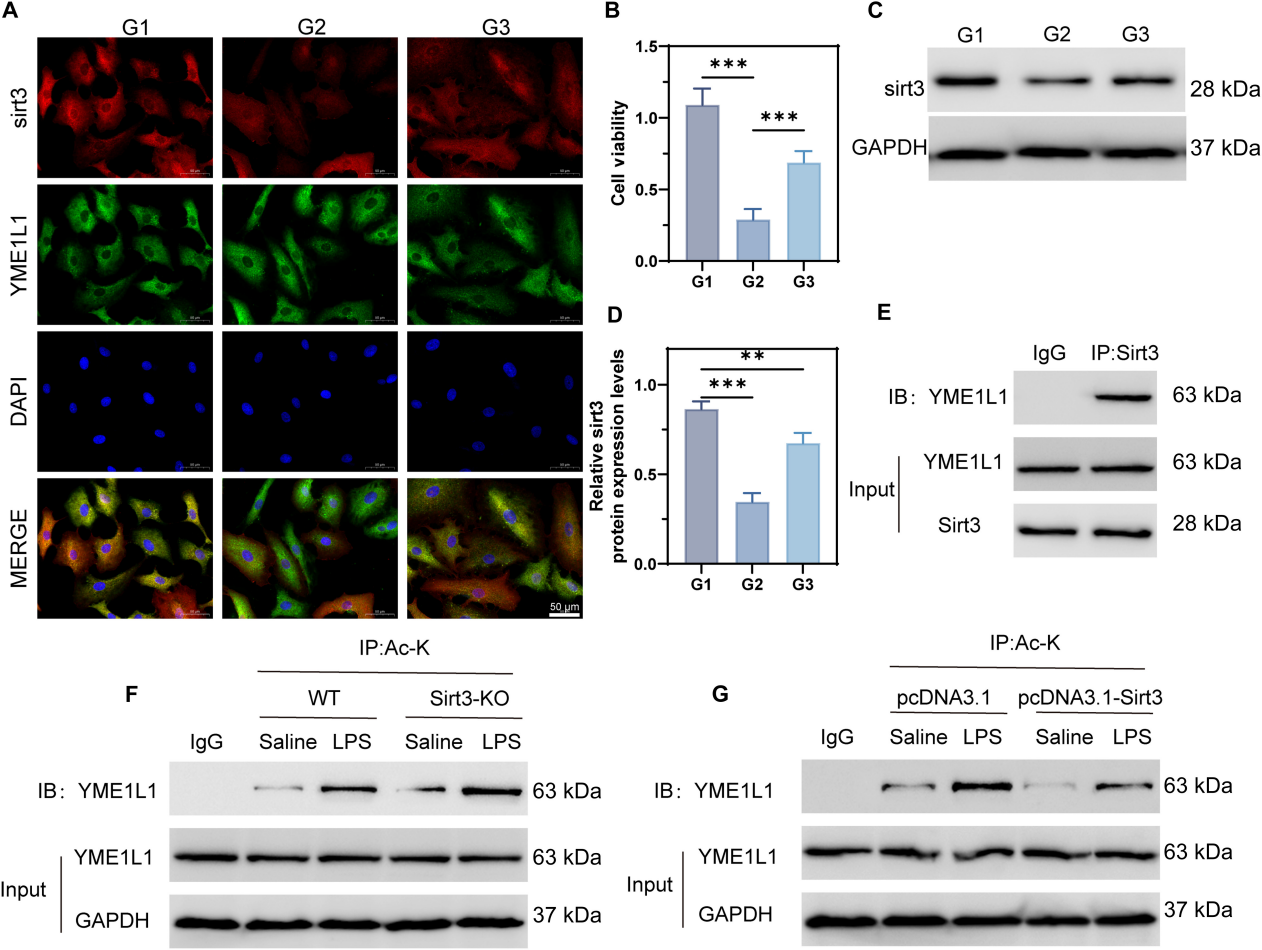
Sirt3 regulates the deacetylation process of YME1L1. (A) Immunofluorescence staining revealed colocalization of Sirt3 and YME1L1. (B) CCK-8. (C and D) WB. (E) Immunoprecipitation assay for Sirt3–YME1L1 interaction. (F) Representative immunoprecipitation (IP) results of the acetylation level ofYME1L1 in renal tissues and semiquantification of these results. (G) Representative IP results of the acetylation level of YME1L1 in HK-2 cells and semiquantification of these results. **P < 0.01, ***P < 0.001.

To further elucidate the mechanism by which Sirt3 and its downstream target YME1L1 function in AKI, relevant assays including IF, WB, and Co-IP were performed. The experimental results are shown in Fig. 7. Initially, HK-2 cells subjected to LPS-induced inflammation were divided into the following groups: G1, Control group; G2, LPS group; and G3, BPNSs@CORT@Raw264.7@(KKEEE)₃K group. The colocalization of Sirt3 and YME1L1 was assessed by IF. As shown in Fig. 7A, both Sirt3 and YME1L1 were expressed in the mitochondria of HK-2 cells. IF staining revealed colocalization of Sirt3 and YME1L1 in normally cultured HK-2 cells. However, LPS treatment attenuated this colocalization. Treatment with BPNSs@CORT@Raw264.7@(KKEEE)₃K increased Sirt3 expression but decreased YME1L1 expression. Subsequently, cell viability was measured using the CCK-8 assay. The results presented in Fig. 7B demonstrated that BPNSs@CORT@Raw264.7@(KKEEE)₃K effectively ameliorated LPS-induced cell damage. Sirt3 protein levels were examined, and as illustrated in Fig. 7C and D, treatment with BPNSs@CORT@Raw264.7@(KKEEE)₃K increased Sirt3 expression. To investigate whether Sirt3 regulates OPA1-mediated mitochondrial fusion by deacetylating YME1L1 in LPS-treated renal tubular epithelial (HK-2) cells, the protein level of YME1L1 and its interaction with Sirt3 were evaluated. WB and IP results indicated that in a mouse model of LPS-induced septic AKI, the total expression level of YME1L1 showed no significant change compared to the control group. However, its acetylation level was increased, and Sirt3 deficiency further enhanced YME1L1 acetylation (Fig. 7F). Furthermore, in HK-2 cells, the total expression level of YME1L1 also remained largely unchanged, but its acetylation level was elevated upon LPS stimulation. Overexpression of Sirt3 reduced the acetylation level of YME1L1 (Fig. 7G). IP results further confirmed an interaction between Sirt3 and YME1L1 in HK-2 cells (Fig. 7E). These findings suggest that Sirt3 deacetylates YME1L1, thereby confirming that Sirt3 regulates OPA1-mediated mitochondrial fusion via deacetylation of YME1L1, which confers a protective role against LPS-induced injury in tubular epithelial cells (HK-2).

### In vivo experimental validation of therapeutic mechanisms

**Fig. 8.**
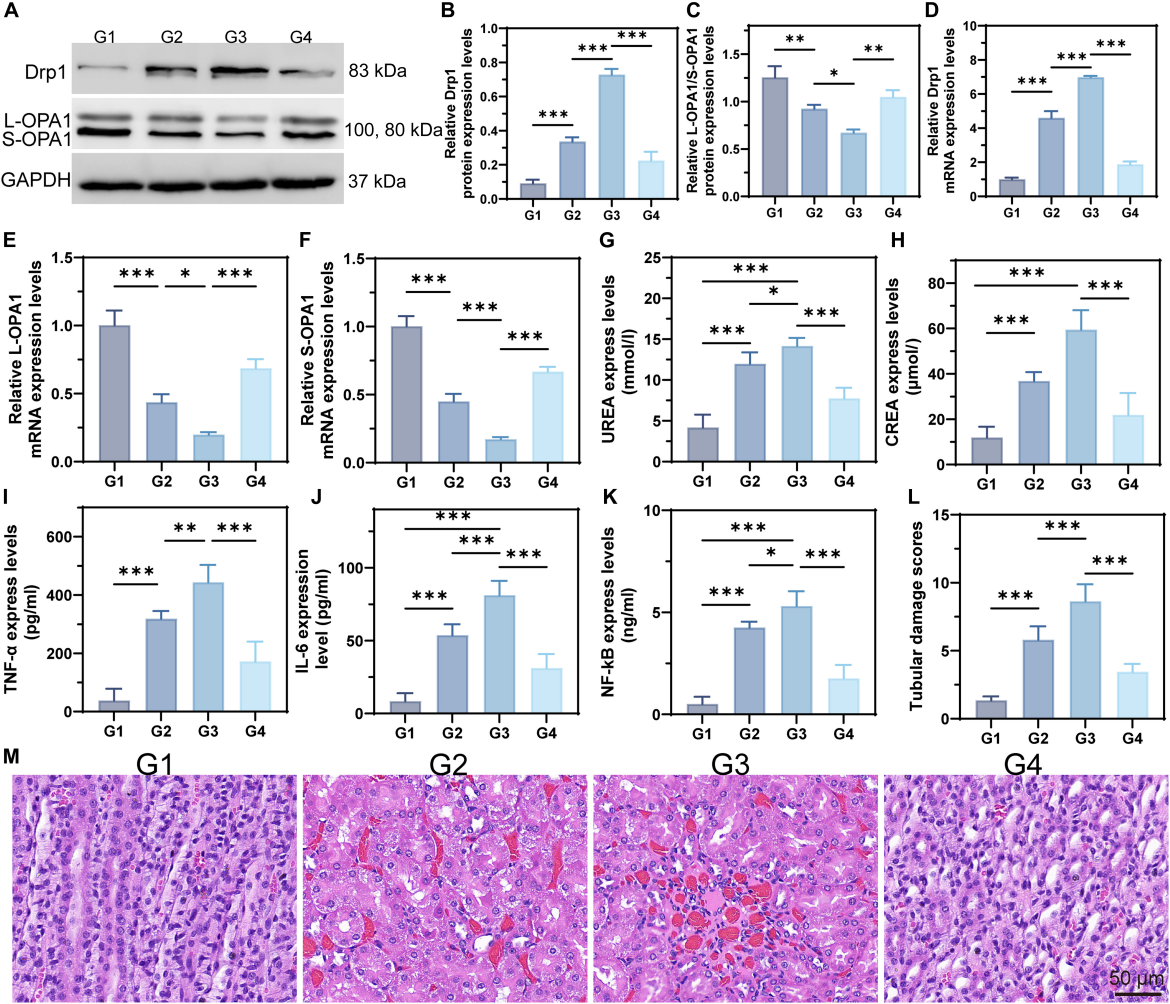
In vivo experimental validation of therapeutic mechanisms. (A to C) WB detection of Drp1, L-OPA1, and S-OPA1 expression. (D to F) RT-PCR detection of Drp1, L-OPA1, and S-OPA1 expression. (G to K) ELISA detection of UREA, CREA, TNF-α, IL-6, and NF-kB expression. (L and M) H&E examination of renal tissue pathology. *P < 0.05, **P < 0.01, ***P < 0.001. G1, Control; G2, Model; G3, si-sirt3; and G4, si-sirt3+BPNSs@CORT@Raw264.7@(KKEEE)_3_K.

Further, through animal experiments, we constructed an SA-AKI animal model and administered BPNSs@CORT@Raw264.7@(KKEEE)₃K treatment. Changes were detected via WB, ELISA, and H&E staining, with experimental results shown in Fig. 8 for groups G1, Control; G2, Model; G3, si-sirt3; and G4, si-sirt3+BPNSs@CORT@Raw264.7@(KKEEE)₃K. First, WB was used to detect Drp1, L-OPA1, and S-OPA1 expression, as shown in Fig. 8A to C, demonstrating that BPNSs@CORT@Raw264.7@(KKEEE)₃K effectively improved mitochondrial function to achieve therapeutic effects. Subsequently, RT-PCR was employed to detect Drp1, L-OPA1, and S-OPA1 expression, with results presented in Fig. 8D to F, which also validated the hypothesis at the molecular level. Next, ELISA kits were used to measure serum UREA, CREA, TNF-α, IL-6, and NF-kB levels in mice, as shown in Fig. 8G to K. The results further indicated that down-regulating Sirt3 expression exacerbated inflammatory levels, while intervention with BPNSs@CORT@Raw264.7@(KKEEE)₃K effectively suppressed the inflammatory response. H&E staining was performed to evaluate renal histopathology, with results shown in Fig. 8L and M. The G2 model group exhibited significant pathological changes, including glomerular enlargement and marked tubular epithelial cell edema. Notably, the G4 group demonstrated effective improvement in renal tissue damage following drug intervention.

### Immunohistochemical analysis of Sirt3, Drp1, YME1L1, BCL-2, Bax, L-OPA1, and S-OPA1 expression

**Fig. 9.**
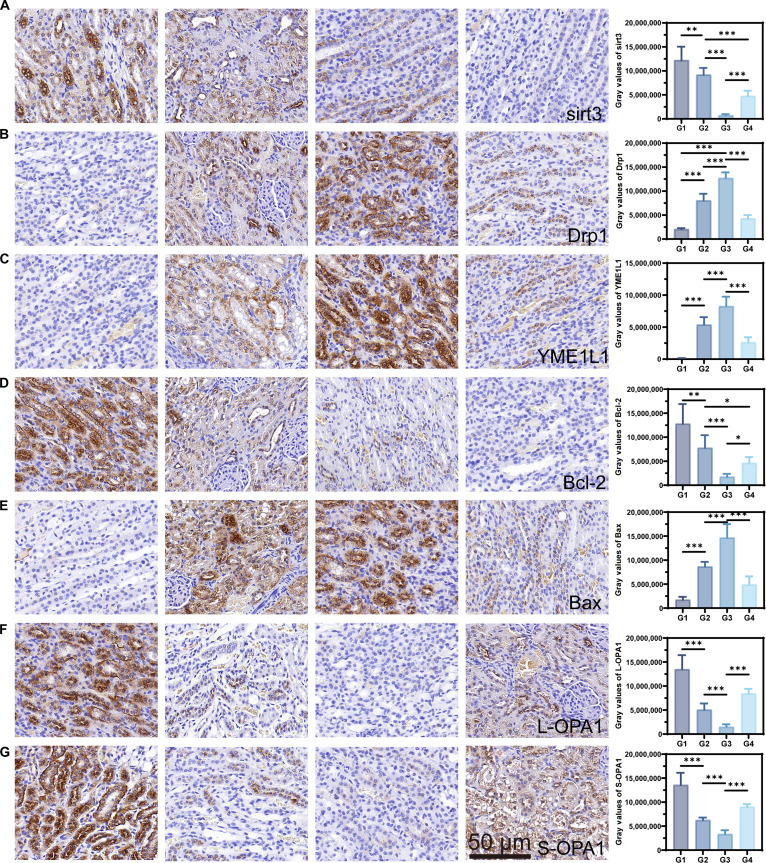
IHC analysis of Sirt3, Drp1, YME1L1, BCL-2, Bax, L-OPA1, and S-OPA1 expression. (A) IHC analysis of Sirt3 expression. (B) IHC analysis of Drp1 expression. (C) IHC analysis of YME1L1 expression. (D) IHC analysis of BCL-2 expression. (E) IHC analysis of Bax expression. (F) IHC analysis of L-OPA1 expression. (G) IHC analysis of S-OPA1 expression. *P < 0.05, **P < 0.01, ***P < 0.001.

To evaluate the effects of BPNSs@CORT@Raw264.7@(KKEEE)₃K on Sirt3, Drp1, YME1L1, BCL-2, Bax, L-OPA1, and S-OPA1 expression, we conducted immunohistochemical (IHC) analysis. The results are shown in Fig. 9 for the G1, Control; G2, Model; G3, si-sirt3; and G4, si-sirt3+BPNSs@CORT@Raw264.7@(KKEEE)₃K groups. As illustrated in Fig. 9A, BPNSs@CORT@Raw264.7@(KKEEE)₃K effectively enhances Sirt3 expression, demonstrating therapeutic efficacy. Additionally, the expression levels of Drp1 (Fig. 9B), YME1L1 (Fig. 9C), and Bax (Fig. 9E) were reduced, while Bcl-2 (Fig. 9D), L-OPA1 (Fig. 9F), and S-OPA1 (Fig. 9G) were up-regulated. These findings suggest that the therapeutic effects are mediated through improved mitochondrial function. Beyond therapeutic efficacy, the biotoxicity of nanodrugs remains a critical evaluation parameter. We conducted histopathological examinations on cardiac, hepatic, splenic, pulmonary, and renal tissues of treated mice (Fig. S1). As shown in Fig. S1A, all organs exhibited normal morphology, demonstrating that BPNSs@CORT@Raw264.7@(KKEEE)₃K nanosheets caused no significant toxicity. Since blood parameters sensitively reflect organ dysfunction, we collected serum samples and analyzed biochemical indicators including ALT, AST, TP, GLB, CK, and LDH. Experimental results (Fig. S1B) revealed that all parameter values remained within normal ranges, indicating that BPNSs@CORT@Raw264.7@(KKEEE)₃K nanosheets effectively reversed septic kidney injury-induced alterations in hematological parameters. These findings collectively confirm the absence of significant toxic side effects in BPNSs@CORT@Raw264.7@(KKEEE)₃K nanosheets. Finally, we conducted survival rate assessments in mice. In an SA-AKI mouse model, the 72-h survival rate of mice in the G5 group [BPNSs@CORT@Raw264.7@(KKEEE)₃K] was significantly higher than that in the G2 group (Model) (Fig. S2) (*P* < 0.01). Furthermore, we conducted pharmacokinetic experiments on pure CORT and BPNSs@CORT@Raw264.7@(KKEEE)₃K alone. The experimental results are shown in Tables S5 and S6 and Fig. S3, demonstrating that the blood concentration of pure CORT was significantly lower than that of BPNSs@CORT@Raw264.7@(KKEEE)₃K. We also observed that pure CORT was rapidly cleared from the system over time. Furthermore, pharmacokinetic parameters calculated based on the fitted equations revealed that after bionic modification of the cell membrane, the elimination rate (K10) of BPNSs@CORT@Raw264.7@(KKEEE)₃K nanoparticles in blood was reduced. Additionally, the half-life of BPNSs@CORT@Raw264.7@(KKEEE)₃K nanoparticles was slightly prolonged, with lower clearance rate and higher mean retention time. For instance, the blood clearance rate of BPNSs@CORT@Raw264.7@(KKEEE)₃K nanoparticles was 0.03 μg/(ng/ml)/h, with an average retention time of approximately 5.5 h, which was longer than that of single CORT (4.5 h) (Tables S5 and S6). These findings further demonstrate that bionic modification of the cell membrane can effectively enhance the circulation time of CORT, thereby improving its pharmacokinetics to some extent.

### Transcriptome sequencing data

**Fig. 10.**
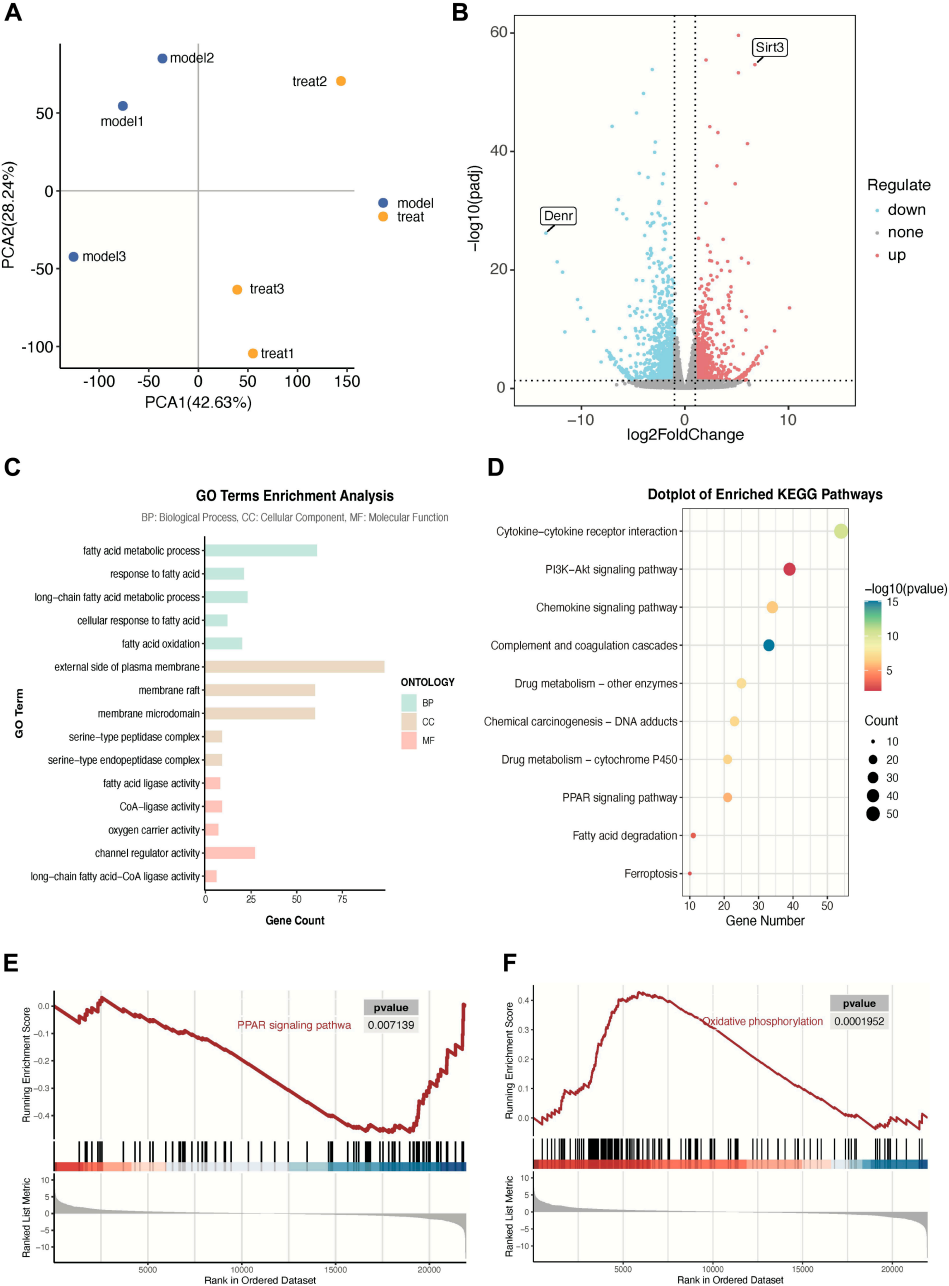
Nanomedicine reverses the pathological state of AKI through transcriptional reprogramming and activates the Sirt3 pathway. (A) Principal component analysis (PCA) demonstrates a significant separation between the nanomedicine-treated group and the SA-AKI model group at the overall transcriptome level, indicating that the treatment induced systemic transcriptional reprogramming. (B) Volcano plot of differentially expressed genes (adjusted *P* value < 0.05, |log_2_FC| > 1). The key gene *Sirt3* was significantly up-regulated (highlighted by a red dot). Dashed lines represent the significance thresholds. (C) Bar graph of Gene Ontology (GO) functional enrichment analysis. The differentially expressed genes were enriched in distinct biological processes. (D) Bubble chart of KEGG pathway enrichment analysis. Following nanomedicine treatment, significant enrichment was observed in inflammation- and immune-related pathways such as “Complement and coagulation cascades” and “Cytokine-cytokine receptor interaction”, as well as in metabolic and protective pathways including the “PPAR signaling pathway” and “Fatty acid degradation”. The bubble size represents the number of enriched genes, and the color indicates the enrichment significance. (E and F) Gene set enrichment analysis (GSEA) plots. Unbiased GSEA validation revealed that the “PPAR signaling pathway” gene set (E) and the “Fatty acid oxidation” gene set (F) were significantly enriched after nanomedicine treatment (*P* value < 0.05). The enrichment score (ES) curve is shown in the middle of each plot, with the distribution of genes in the ranked list displayed at the bottom.

Furthermore, we analyzed the transcriptomic sequencing data (Treatment vs. Model) to directly untangle which pathways and genes were targeted by the nanomaterial’s therapeutic effects (Fig. 10). Given that Sirt3-mediated mitochondrial dysfunction is undoubtedly central, the analysis was specifically focused on this aspect. Principal component analysis (Fig. 10A) revealed a clear separation between samples from the nanodrug-treated group and the SA-AKI model group at the transcriptomic level, indicating that nanodrug treatment induced significant transcriptional reprogramming. The volcano plot (Fig. 10B) showed that key genes, including Sirt3, were significantly up-regulated after nanodrug treatment, demonstrating successful targeting and up-regulation of Sirt3 expression by the nanodrug. Gene Ontology (GO) enrichment analysis (Fig. 10C) indicated that the differentially expressed genes were primarily enriched in biological processes such as fatty acid metabolism and oxidative stress response. KEGG pathway enrichment analysis (Fig. 10D) revealed that metabolic pathways, including the PPAR signaling pathway and fatty acid degradation, were activated following nanodrug treatment, while pathways related to inflammation and apoptosis were suppressed. Gene set enrichment analysis (Fig. 10E and F) demonstrated significant enrichment of the PPAR signaling pathway after nanodrug treatment, confirming its activation. Significant enrichment of the fatty acid oxidation pathway was also observed, providing further evidence for the regulatory role of the nanodrug in energy metabolism.

## Discussion and Conclusion

### Discussion

AKI caused by sepsis is a common clinical critical condition, with its high mortality closely linked to the lack of specific therapeutic approaches [44]. Current treatment strategies primarily rely on supportive therapies such as fluid resuscitation and renal replacement therapy, yet fail to effectively reverse the core pathological mechanisms of mitochondrial dysfunction and inflammatory cascade reactions. Although studies have explored the role of Sirt3 in renal protection, the mechanism by which it regulates mitochondrial dynamics through deacetylation of YME1L1 remains unclear. Existing nanodelivery systems also face challenges such as insufficient targeting and limited therapeutic efficacy. Liposomal nanoparticles developed in some studies can alleviate inflammation but fail to simultaneously repair mitochondrial damage [45–48]. While Sirt3’s protective role in ischemia–reperfusion AKI has been confirmed, its regulation of YME1L1 in septic models remains unexplored. These limitations highlight the urgent need to develop novel multitarget therapeutic strategies. This study achieved 3 major breakthroughs by constructing macrophage membrane-encapsulated BPNSs loaded with CORT receptor agonist and modified with a targeted peptide (KKEEE)₃K to form a nano-complex [BPNSs@CORT@Raw264.7@(KKEEE)₃K]. First, the nanosystem significantly enhanced drug uptake concentration by leveraging the natural inflammatory chemotaxis of macrophage membranes and the renal targeting of (KKEEE)₃K. Second, the complex activated Sirt3 via CORT, promoting YME1L1 deacetylation to restore mitochondrial fission/fusion balance, while BPNSs directly scavenged ROS, creating a synergistic “mechanism repair-inhibiting oxidative stress” effect. Third, the study demonstrated the pivotal role of the Sirt3–YME1L1 axis in sepsis models for the first time, with molecular mechanisms clarified through siRNA knockdown and Co-IP experiments. Animal experiments further showed that the treatment significantly reduced serum creatinine/urea levels (*P* < 0.01) and improved renal pathology, outperforming traditional single-target strategies. Future research could optimize peptide design (e.g., introducing RGD sequences) to enhance renal specificity. This study not only provides a novel therapeutic strategy for SA-AKI but also lays theoretical foundations for the development of nano-metabolic regulation combined therapies.

### Conclusion

This study successfully developed a novel nano-complex [BPNSs@CORT@Raw264.7@(KKEEE)₃K] by encapsulating CORT receptor agonists on macrophage membrane-coated BPNSs with a targeted peptide (KKEEE)₃K. We systematically investigated its therapeutic effects and molecular mechanisms in SA-AKI. Through in vitro and in vivo experiments, we demonstrated that the nano-complex effectively improves mitochondrial dysfunction and inflammatory responses via the Sirt3-mediated YME1L1 deacetylation pathway, providing a novel therapeutic strategy for SA-AKI. In vitro experiments showed that the nano-complex significantly increased survival rates of renal tubular epithelial cells (HK-2) and reduced expression of inflammatory factors (TNF-α, IL-6, and NF-κB), while restoring mitochondrial dynamics by regulating the balance between Drp1 and OPA1. IF and Co-IP experiments further confirmed the interaction between Sirt3 and YME1L1, clarifying the pivotal role of the Sirt3–YME1L1 axis in SA-AKI. In animal models, the nano-complex significantly improved renal function indicators (CREA and UREA) and reduced pathological kidney damage, revealing its potential to regulate mitochondrial-related genes and pathways. In summary, this study not only provides a new nanodrug candidate for SA-AKI treatment but also opens new avenues for intervention strategies targeting mitochondrial dysfunction-related diseases. Future work will focus on preclinical translation and personalized treatment development.

## Ethical Approval

All animal experiments were conducted in strict accordance with the guidelines of the Institutional Animal Care and Use Committee (IACUC)and were approved under protocol number DWSY-23037341. All procedures adhered to the National Institutes of Health (NIH) *Guide for the Care and Use of Laboratory Animals* (8th edition, 2011). Efforts were made to minimize animal suffering and reduce the number of animals used.

## Data Availability

A data availability statement is not applicable to this manuscript.
