# Supplementary material for: Targeted Sirtuin 3 Activation by Biomimetic Black Phosphorus Nanosheets Mitigates Sepsis-Induced Acute Kidney Injury through Yeast Mitochondrial Escape 1-Like 1 Deacetylation
Source: Biomater Res. 2026 Jun 29;30:0379. doi: 10.34133/bmr.0379 (PMC13311258; doi:10.34133/bmr.0379)
Supplement: Supplementary 1 — Figs. S1 to S3 Tables S1 to S6 [file bmr.0379.f1.zip › Supplementary Figure.docx]

**Supporting information**

**For**

Targeted Sirtuin 3 Activation by Biomimetic Black Phosphorus Nanosheets Mitigates Sepsis-Induced Acute Kidney Injury through Yeast Mitochondrial Escape 1-Like 1 Deacetylation


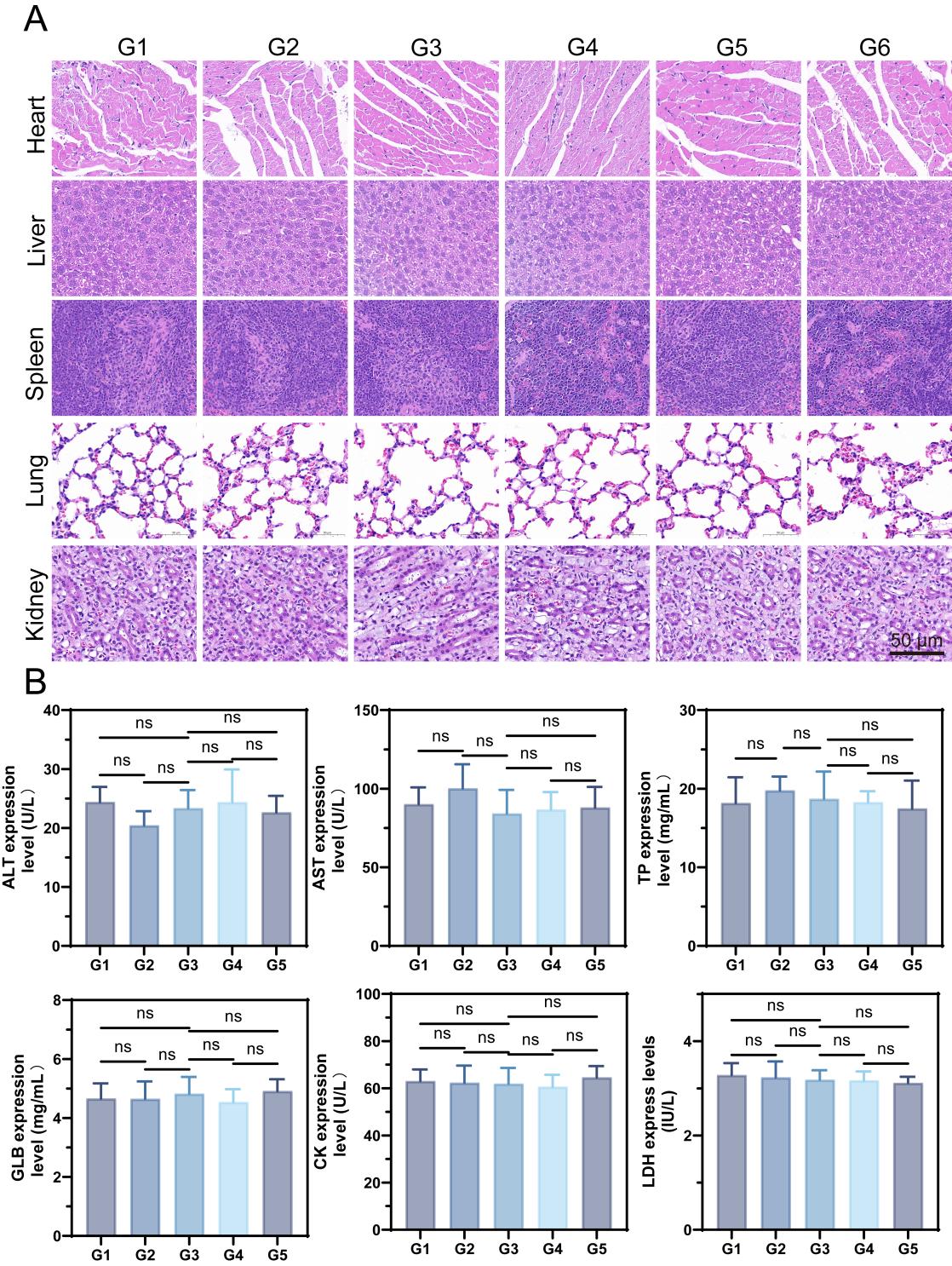


**Figure S1 In vivo toxicity test.**(A)The heart, liver, spleen, lung and kidney were examined pathologically.(B)Elisa detection of ALT,AST,TP,GLB,CK,LDH expression.****P* < 0.05, ***P* < 0.01, ****P* < 0.001.**


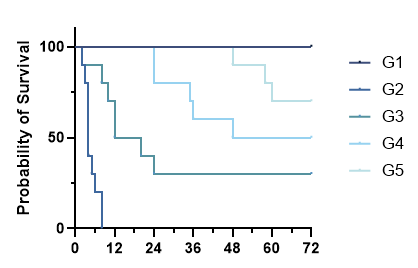


**Figure S2 Mouse survival rate experiment.**G1. Control; G2. Model; G3. BPNSs@CORT; G4. BPNSs@CORT@Raw264.7; G5. BPNSs@CORT@Raw264.7@(KKEEE)₃K.


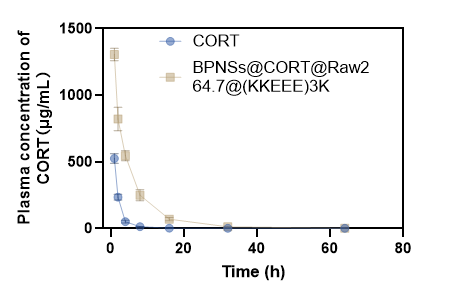


**Figure S3 Pharmacokinetics of CORT, BPNSs@CORT@Raw264.7@ (KKEEE)₃K.**
