# Supplementary material for: Targeted Sirtuin 3 Activation by Biomimetic Black Phosphorus Nanosheets Mitigates Sepsis-Induced Acute Kidney Injury through Yeast Mitochondrial Escape 1-Like 1 Deacetylation
Source: Biomater Res. 2026 Jun 29;30:0379. doi: 10.34133/bmr.0379 (PMC13311258; doi:10.34133/bmr.0379)
Supplement: Supplementary 1 — Figs. S1 to S3 Tables S1 to S6 [file bmr.0379.f1.zip › Supplementary Tables.docx]

**Supplementary Table**

| Antibodies | Dilution (application) |
| --- | --- |
| Bcl-2 | 1:50 (IF) |
| Bax | 1:100 (IF) |
| Goat anti-Rat IgG (H+L) Cross-Adsorbed Secondary Antibody, Alexa Fluor™ 488 | 1:2000 (IF) |
| Goat Anti-Rabbit IgG H&L (Alexa Fluor® 594) | 1:500 (IF) |

**Table S1 Immunofluorescence antibody information.**

| Antibodies | Dilution (application) |
| --- | --- |
| Drp1  OPA1  Sirt3  Bax  Bcl-2  YME1L1 | 1:5000 (WB)  1:1000 (WB)  1:2000 (WB)  1:1000 (WB)  1:1000 (WB)  1:5000 (WB) |
| GAPDH | 1:10000 (WB) |
| Goat Anti-Rabbit IgG H&L(HRP) | 1:20000 (WB) |
| Goat Anti-Mouse IgG H&L(HRP) | 1:20000 (WB) |

**Table S2 WB Antibody Information.**

| Batch | Encapsulation rate（%） |
| --- | --- |
| 1 | 86.51 |
| 2 | 89.54 |
| 3 | 82.36 |
| Average value | 86.14 ± 3.60 |

**Table S3 BPNSs@CORT@Raw264.7@ (KKEEE)₃K encapsulation rate.**

| Batch | Drug loading capacity（%） |
| --- | --- |
| 1 | 17.89 |
| 2 | 16.81 |
| 3 | 15.68 |
| Average value | 16.79 ±1.11 |

**Table S4 BPNSs@CORT@Raw264.7@ (KKEEE)₃K drug loading capacity.**

| Parameter | Unit | Value |
| --- | --- | --- |
| K10 | 1/h | 1.25 |
| K12 | 1/h | 0.78 |
| K21 | 1/h | 0.17 |
| t1/2α | h | 0.33 |
| t1/2β | h | 6.97 |
| C0 | ng/ml | 4751.73 |
| V | (μg)/(ng/ml) | 0.08 |
| CL | (μg)/(ng/ml)/h | 0.10 |
| V2 | (μg)/(ng/ml) | 0.39 |
| CL2 | (μg)/(ng/ml)/h | 0.07 |
| AUC 0-t | ng/ml*h | 3322.56 |
| AUC 0-inf | ng/ml*h | 3812.56 |
| AUMC | ng/ml*h^2 | 17302.25 |
| MRT | h | 4.54 |
| Vss | μg/(ng/ml) | 0.48 |

**Table S5 Pharmacokinetic parameters of CORT.**

| Parameter | Unit | Value |
| --- | --- | --- |
| K10 | 1/h | 0.49 |
| K12 | 1/h | 0.91 |
| K21 | 1/h | 0.54 |
| t1/2α | h | 0.39 |
| t1/2β | h | 4.74 |
| C0 | ng/ml | 7188.21 |
| V | (μg)/(ng/ml) | 0.06 |
| CL | (μg)/(ng/ml)/h | 0.03 |
| V2 | (μg)/(ng/ml) | 0.09 |
| CL2 | (μg)/(ng/ml)/h | 0.05 |
| AUC 0-t | ng/ml*h | 14456.34 |
| AUC 0-inf | ng/ml*h | 14808.18 |
| AUMC | ng/ml*h^2 | 82062.27 |
| MRT | h | 5.54 |
| Vss | μg/(ng/ml) | 0.15 |

**Table S6 Pharmacokinetic parameters of BPNSs@CORT@Raw264.7@ (KKEEE)₃K.**
